# The Perfect Condition for the Rising of Superbugs: Person-to-Person Contact and Antibiotic Use Are the Key Factors Responsible for the Positive Correlation between Antibiotic Resistance Gene Diversity and Virulence Gene Diversity in Human Metagenomes

**DOI:** 10.3390/antibiotics10050605

**Published:** 2021-05-20

**Authors:** Célia P. F. Domingues, João S. Rebelo, Joël Pothier, Francisca Monteiro, Teresa Nogueira, Francisco Dionisio

**Affiliations:** 1 cE3c—Centre for Ecology, Evolution and Environmental Changes, Faculdade de Ciências, Universidade de Lisboa, 1749-016 Lisboa, Portugal; celiapfd@hotmail.com (C.P.F.D.); joaorebelo_4@hotmail.com (J.S.R.); fsmonteiro@fc.ul.pt (F.M.); 2 INIAV—National Institute for Agrarian and Veterinary Research, Bacteriology and Mycology Laboratory, 2780-157 Oeiras, Portugal; 3 Atelier de Bioinformatique, ISYEB, UMR 7205 CNRS MNHN UPMC EPHE, Muséum National d’Histoire Naturelle, CP 50, 45 rue Buffon, F-75005 Paris, France; joel.pothier@mnhn.fr

**Keywords:** antibiotic resistance, virulence, microbiome, metagenomics, human gut, computer simulation

## Abstract

Human metagenomes with a high diversity of virulence genes tend to have a high diversity of antibiotic-resistance genes and vice-versa. To understand this positive correlation, we simulated the transfer of these genes and bacterial pathogens in a community of interacting people that take antibiotics when infected by pathogens. Simulations show that people with higher diversity of virulence and resistance genes took antibiotics long ago, not recently. On the other extreme, we find people with low diversity of both gene types because they took antibiotics recently—while antibiotics select specific resistance genes, they also decrease gene diversity by eliminating bacteria. In general, the diversity of virulence and resistance genes becomes positively correlated whenever the transmission probability between people is higher than the probability of losing resistance genes. The positive correlation holds even under changes of several variables, such as the relative or total diversity of virulence and resistance genes, the contamination probability between individuals, the loss rate of resistance genes, or the social network type. Because the loss rate of resistance genes may be shallow, we conclude that the transmission between people and antibiotic usage are the leading causes for the positive correlation between virulence and antibiotic-resistance genes.

## 1. Introduction

Since the 1940s, antibiotics are widely used in human and animal health to cure infections and as growth promoters in livestock and agriculture [1]. The initial optimism has been gradually declining as several factors have contributed to the decrease in the effectiveness of antibiotics in the so-called antibiotic crisis. Namely, extensive use in livestock and fish farming, non-adherence to antibiotic treatment and over-prescription, poor infection control in hospitals and health care settings, poor sanitation and environmental contaminations, together with the lack of development of new antibiotics, have contributed to antibiotics liability [2].

As an incredible example of Darwinian selection, bacteria worldwide have gradually become resistant to several antibiotics. Antibiotics select for resistant clones and prompt the emergence of new ones by triggering mutation rate and horizontal gene transfer, e.g. through the activation of the SOS response [3]. The spread of resistance has terrible consequences. For example, there were 875,500 disability-adjusted life-years and more than 33,000 deaths in European Economic Area due to antibiotic resistance in 2015 [4]. Worldwide, at least 700,000 people die annually due to drug-resistant diseases [5]. Because of the adaptive power of bacteria and the massive use of antibiotics, we are now in the so-called ‘post-antibiotic’ era [6,7,8].

The human microbiota is the set of all the microorganisms that inhabit the human body. It is often very complex, comprising both pathogenic and non-pathogenic bacteria. The human microbiome is the catalog of human microbes and their genes [9]. Microorganisms of the microbiome comprise about 3.8 × 10^13^ bacterial cells [10], spanning thousands of taxa. They colonize the body’s surfaces and biofluids, including tissues, such as skin, mucosa and, importantly, the gastrointestinal tract [9].

Virulence factors are proteins that help bacteria colonizing a host or biome. According to Liu and colleagues, “Virulence factors refer to the properties (i.e., gene products) that enable a microorganism to establish itself on or within a host of a particular species and enhance its potential to cause disease. Virulence factors include bacterial toxins, cell surface proteins that mediate the bacterial attachment, cell surface carbohydrates and proteins that protect a bacterium, and hydrolytic enzymes that may contribute to the pathogenicity of the bacterium” [11]. These traits are easily spread in bacterial populations or microbiomes by horizontal gene transfer, which can potentially convert mutualistic or commensal bacteria into pathogens able to progress into new tissues, triggering an infectious disease [12]. That is the case of *Escherichia coli* bacterial species, which can have many different pathotypes, ranging from those responsible for mild infections to those that can inflict severe disease or even death. For example, the extraintestinal pathogenic strains can circulate in the blood and invade the brain endothelial cells, thus crossing the blood-brain barrier—when they harbor the *ibe* gene [13] that can be encoded in plasmids, such as p157 or p026_1 [14]. Hence, horizontal gene transfer plays a significant role in keeping bacterial social behavior within the consortium [15], during infection, when different bacteria contribute to the synthesis of virulence traits.

There is a positive correlation between antibiotic resistance genes’ diversity and virulence genes’ diversity across human gut microbiomes [16]. This correlation is in line with the evidence published on the co-occurrence of virulence and antibiotic resistance traits in individual bacterial genomes [17,18,19]. Could this positive correlation result from administering antibiotics in sick people due to bacterial infections, eventually selecting bacteria encoding virulence and resistance determinants simultaneously? The administration of antibiotics to clear drug-sensitive pathogenic bacteria may select a few clones that, by chance, just acquired a resistance determinant, such as a spontaneous chromosomal mutation, or resistance genes through horizontal gene transfer, contributing to the co-selection of virulence and resistance genes. Eventually, this procedure could lead to a positive correlation between the diversity of virulence and resistance genes. Yet, two facts undermine this prediction: (i) many non-pathogenic bacteria of the microbiome may also become drug-resistant through chromosomal mutations or the acquisition of resistance genes by horizontal gene transfer; (ii) antibiotics are often used as growth factors of animals, not for treatments. Indeed, many non-pathogenic (mutualistic or commensal) strains and species are undoubtedly affected, which has been demonstrated [20,21,22]. Therefore, an explanation for the positive correlation mentioned above is still missing.

Several factors may impact genes’ diversity. In the case of antibiotic resistance genes, taking a certain antibiotic selects the genes that confer resistance to that specific drug. Simultaneously, the same antibiotic also eliminates other genes conferring resistance to other antibiotics possibly present in the bacteria of a metagenome. Consequently, even if antibiotic intake may increase the number of genes conferring resistance to that specific drug in the metagenome, the diversity of drug resistance genes decreases. Such a decrease in diversity is a special case of the gut microbiota dysbiosis caused by antibiotic administration [23,24]. For the same reason, antibiotic administration also decreases the diversity of virulence genes.

In the absence of antibiotics, resistance determinants impose a fitness cost, both in the case of resistance mutations [25] and plasmids encoding resistance genes [26]. By growing slower than sensitive clones, resistant clones are prone to be displaced by them. Therefore, in time, metagenomes may lose resistance genes.

Humans in close contact may share their microorganisms, thus enriching their microbiomes with new bacteria (either commensal, mutualistic, or pathogenic) and new genes, including those encoding for virulence and antibiotic resistance. Indeed, human microbiomes are more similar among humans living together, irrespective of the genetic relatedness, suggesting that transmission is a critical factor of the microbiome constitution [27,28]. Microbes’ transmission from mother to child by skin contact, breastfeeding and kissing, is one of the best-studied examples of very close contact and microbiome enrichment [29,30,31,32,33]. These studies suggest that bacteria in human microbiomes can have a shared exposure or result from person-to-person transfer on the social network [34]. When someone in the community takes antibiotics, there can be selection for resistance genes in his/her metagenome and this individual may become a source of resistance genes to his/her contacts in the network. The dissemination of bacteria and their genes may enrich the human microbiome of his/her contacts with virulence and resistance genes. Therefore, transmission between people should play a role in keeping the correlation between resistance and virulence genes’ diversity.

Many individual-based models assume simple contact patterns, such as regular lattices (e.g., Reference [35]) or random networks where all individuals can infect all others. Still, natural populations will almost certainly follow none of these two model types of microorganisms’ spread. Most natural populations have a structure between these two cases [36]. In the simulations performed here, we assume structured populations organized in three types of contact networks: regular, random, and small-world. In this work model, each node of the network corresponds to a person (its metagenome).

Regular networks present both a large clustering degree (defined as the probability that two nodes are connected, given that they share a close neighbor) and a large characteristic path length (defined as the mean of the minimum distance between all pairs of vertices in the network). In contrast to regular networks, completely random networks present both a low clustering coefficient and a low characteristic path length. However, none of the models are adequate to describe specific systems, e.g., the structure of human populations. A few alternative models have been suggested to describe these systems better. Small-world networks have been highly successful [37,38]. These networks reveal a small mean path length, like random graphs, but a high clustering coefficient, as regular lattices. Epidemiologists have found these small-world properties in the spread of infectious diseases and epidemics [36,39] and social interactions [40].

This work aims to find the key factors leading to the positive correlation between the diversity of virulence and antibiotic resistance genes observed across human metagenomes [16]. To this end, we have developed a computer model of a hypothetical network of human beings that share their microbiomes (bacteria and genes). We simulated the transfer of bacterial pathogens, antibiotic resistance and virulence genes in a human-to-human transmission network.

We analyzed the impact of several variables, including the network structure, on the resistance and virulence diversities’ final correlation. We show that a positive correlation between the diversity of antibiotic resistance coding genes and those coding for virulence emerges whenever the gene transmission rate between individuals is higher than the probability that metagenomes lose resistance genes, irrespectively of all the other variables tested. This simple rule explains the positive correlation between virulence genes’ diversity and antibiotic resistance genes’ diversity. Moreover, the diversity of antibiotic resistance and virulence genes is high in individuals who have passed a long time since they took antibiotics and minimal for those who just took antibiotics.

## 2. Methods

### 2.1. Building the Human Network 

**Figure 1:**
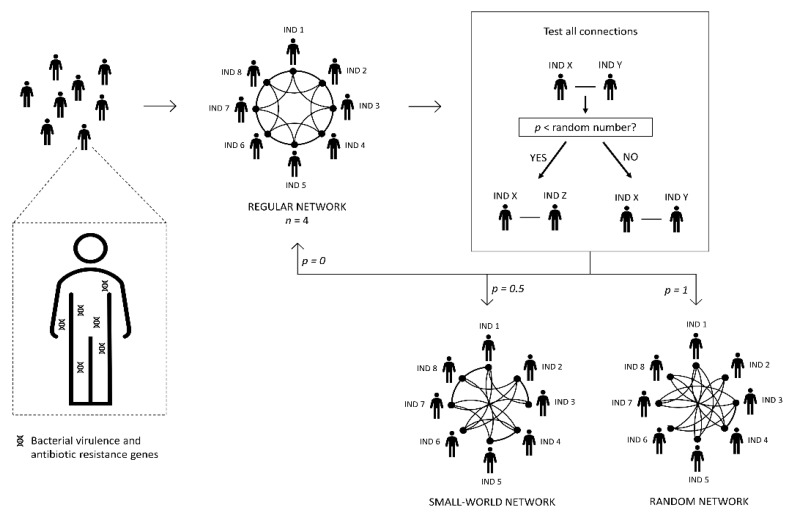
Each human being in a community is colonized by bacteria (as an integral part of the microbiome). Some bacteria contain antibiotic resistance genes and/or virulence genes. These bacteria can be transmitted from one person to another, which includes the passage of resistance and/or virulence genes that they may contain. All the individuals in the community are organized in a network of contacts. In the regular network, each individual is linked to other four individuals (the four closest individuals; *n* = 4). The other two types of network were constructed following Watts and Strogatz method [37]. From the regular network, each connection has a probability p to change, that is, each individual can be reconnected to another one in the network. Therefore, the value of *p* defines the type of network: (i) *p* = 0 the network remains regular; (ii) *p* = 0.5 is a small-world network; (iii) *p* = 1 the network becomes random.

We simulated a network (Figure 1) where each node represents a person or, more precisely, a person’s metagenome (in this case, bacteria and bacterial genes). To simplify language, from now on, we refer to these human metagenomes in the network of contacts as a person or people, meaning a person’s metagenome or people’s metagenomes, respectively. The edges represent possible transmission avenues of microorganisms.

We built the social contact network following the Watts and Strogatz method [37]. In a regular network, each node links to the *n* nearest nodes. In non-regular networks, each node’s link has a certain probability *p* of being reconnected to another randomly chosen node. The parameter *p* represents the probability of each connection to be modified. We defined the network type by the value assigned to the parameter *p* (for example, a regular network when *p* = 0, whereas *p* = 1 results in a random network). Unless noted, we performed simulations with *p* = 0.5 and *n* = 4.

### 2.2. The Metagenome, Pathogenic Bacteria, and Antibiotic Administration

The computational model considers a hypothetical situation of a contact network made of healthy people that share bacteria from their skin, oropharyngeal, or gut microbiomes. Each human being shares his/her bacteria, including non-housekeeping genes that are accessory and yet important to the environmental adaptation, such as virulence and antibiotic resistance.

The model considers the transmission of bacterial pathogens (able to cause infections) and virulence and antibiotic resistance genes. We focused on the diversity of functions encoded, irrespectively of its copy-number in the metagenome. That means that we are addressing the functional diversity, and thus genotypes, of the metagenome. Accordingly, in the simulations, we consider the presence of gene families (with similar functions) that we refer to as “gene” from now on. We divided resistance genes into groups, each group having the same number of families. Each group represents genes associated with resistance to an antibiotic, encoding all the proteins involved in that mechanism of resistance. Of note, we did not consider resistance to multiple drugs in our simulations. Therefore, there are as many groups as there are antibiotics accounted for in the simulations.

To simulate the flow of bacteria from individuals outside the network or the transmission from sources, such as food or contaminated water, we inserted five different bacterial pathogenic species into random individuals once per cycle. In this model, we only categorize species according to the antibiotic they are susceptible to, as explained below.

We assume that individuals infected by pathogenic bacteria feel sick and take an antibiotic prescribed to that pathogen. The antibiotic selects cells carrying resistance genes by clearing susceptible bacteria. In this work, we assume that each resistance gene present in a metagenomecan be in two different possible levels: in some metagenomes, they are present in low copy number, so we assume that they are not transmissible to other individuals in the network; in other metagenomes, the copy number of resistance genes is high due to the selective pressure of antibiotics to which they were previously submitted. In the latter case, we assume that they are more likely to be transferred from a person to another.

Moreover, upon antibiotic consumption, the following events can occur: (i) elimination of the pathogenic bacteria involved in the infection; and (ii) selection, in the metagenome, of resistance genes belonging to the same group of resistance as the antibiotic used—this means that the copy number of these resistance genes gets so high that they become transferable to other people. Finally, the model also considers that any antibiotic decreases the bacterial diversity of the microbiome. Specifically, the model considers that the antibiotic kills bacteria carrying virulence genes or carrying genes that confer antibiotic resistance but not the one used. Therefore, the computer model assumes that the antibiotic administrated: (iii) eliminates virulence genes with a certain probability; and (iv) eliminates resistance genes associated with other antibiotics with a certain probability—note that we consider that these genes become non-transferable, but they are still present in low copy numbers.

As explained in the introduction, in the absence of antibiotics, resistance determinants confer a fitness cost. Accordingly, the model considers that each metagenome loses specific resistance genes according to a “loss rate” (with this process, these genes become non-transferable) [33].

### 2.3. Algorithm of the Program

**Figure 2:**
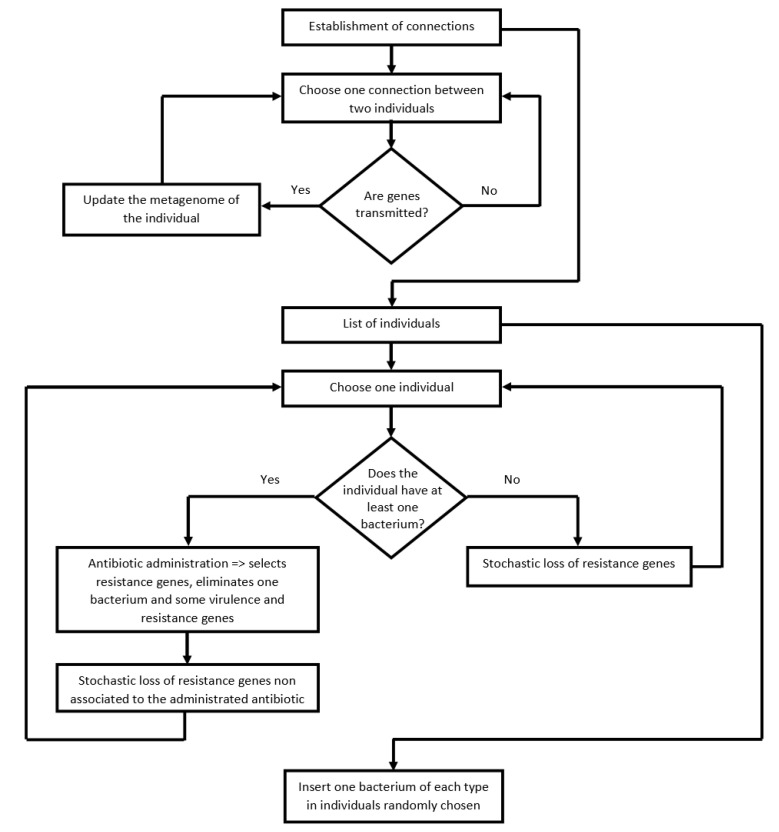
Flowchart of the program. After the network’s construction, the program performs several cycles where, eventually, there is gene transfer between nodes (people). Some individuals get sick and take antibiotics. Some genes are lost due to antibiotic pressure or the fitness cost imposed by resistance genes.

We described the implementation of the procedures in detail in the pseudocode (Table 1; also see the flowchart in Figure 2). All code is available on GitHub https://github.com/cpfdomingues/simulation-code-human-transmission-genes-bacteria (accessed on 4 March 2021).

At the beginning of each simulation, we randomly distribute all bacterial pathogens, all virulence gene families, and all resistance gene families by the population—group of people considered in the network during the simulation. We distribute each virulence gene, each resistance gene, and each bacterial pathogen among 1 to 2% of the population.

Taking the example of virulence genes, we proceed as follows: (i) take a random number between 0.01 (1%) and 0.02 (2%) to obtain the percentage of individuals that should receive a gene of virulence; (ii) convert this percentage into the number of individuals that is going to receive the gene according to the size of the population (% obtained * size of the population); (iii) select randomly as many individuals as those obtained in (ii); (iv) activate this gene in the metagenomes of the selected individuals.

For example, consider a simulation with a network of 1000 individuals and 100 virulence genes. We start with the first virulence gene: (i) suppose we get the number 0.012 randomly; (ii) that means that 0.012 × 1000 = 12 individuals are going to receive the first virulence gene; (iii) we randomly select 12 individuals out of 1000; (iv) we activate that virulence gene in the 12 individuals.

We proceed in this way with all virulence genes, all resistance genes, and all pathogenic bacteria considered in the simulation (Table 2).

To parameterize the model, we performed exploratory simulations, each one composed of several cycles. Parameters that do not influence the correlation sign (Suppl. Tables S3.1–S6.6, S8.1, and S8.2) have a default value shown in Table 2.

The main steps of the program in each cycle are:

(i) Transfer of pathogenic bacteria, virulence, and resistance genes between people (i.e., between linked nodes), according to specific transmission probabilities (Table 2). With this process, the diversity of genes present in the recipient metagenome increases.
(ii) Select people infected by at least one pathogenic bacterium. These people take antibiotics (chosen according to the pathogen). The antibiotic clears the pathogen and selects for resistance genes for the antibiotic used. According to a certain probability (Table 2), the antibiotic also eliminates virulence genes and resistance genes unrelated to the administered antibiotic. Finally, the metagenome loses a few more resistance genes not associated with the antibiotic, according to the loss rate probability (Table 2). The cause of this loss is the fitness cost of resistance genes.
(iii) The metagenomes of people that did not take an antibiotic in this cycle also lose resistance genes according to the loss rate probability (Table 2). As above, this loss is a consequence of the fitness cost imposed by resistance genes on their hosts, which is not happening with virulence genes.
(iv) Add the five bacterial pathogens in five randomly-chosen individuals of the community.

### 2.4. Statistical analysis

We considered that *Y* (diversity of resistance genes) correlates with *X* (diversity of virulence genes), according to:

*Y* = *a*.*X* + b.

In this equation, parameter *a* represents the linear regression slope, while b represents the point at which the line crosses the *y*-axis.

Given the complexity of human interactions, it is paramount to simplify the computer simulations. A simplified model allows us to comprehend the effect of specific factors in our simulations, which would otherwise be extremely difficult to detect. As these simplifications do not allow us to make quantitative inferences, we make qualitative analyses. The focus is always on the correlation or linear regression slope sign between the diversity of virulence and antibiotic resistance genes and whether the correlation is significantly different from zero. Accordingly, the null hypothesis is that there is no correlation between antibiotic resistance genes’ diversity and virulence genes’ diversity. The alternative hypothesis is that there is a correlation between antibiotic resistance genes’ diversity and virulence genes. We define *α* = 1 × 10^−6^, rejecting the null hypothesis if *P*-value < *α*.

We performed the data analyses described above, and the Student’s *t*-tests (see Supplementary Information) with R—version 3.5.1 [41].

## 3. Results

### 3.1. The Number of Diseases and the Probability of Transmission

This work aims to understand the positive correlation between antibiotic resistance genes’ diversity and virulence genes in metagenomes across human populations observed by Escudeiro et al. (2019) (Reference [16]). As explained in the Methods section, we assumed that people interact with each other in a network of connections that enables the flow of bacterial pathogens, antibiotic-resistance and virulence genes. In the simulations, five different pathogenic bacteria circulate between linked people. When pathogenic bacteria infect an individual, that person takes an antibiotic. The antibiotic clears, not only the bacterial pathogen, but also removes a fraction of virulence and resistance genes.

A priori, the pathogen transmission probability could have any value. However, if this value is too high or too low, we reach unrealistic stages. For too high transmission probabilities of pathogens, several people become infected by more than two pathogens simultaneously, which may be unrealistic. For too low transmission probabilities, the pathogen is extinct from the system (because sick people eliminate them with antibiotics before the pathogen has the opportunity to go to another host). Given the importance of this parameter, we must calibrate its value to avoid these situations. Therefore, we started this study searching for the parameters that (i) led individuals to have no more than two pathogenic species simultaneously at a given cycle; and (ii) end each cycle with at least a pathogen in the population.

Accordingly, we performed simulations with different bacterial pathogen transmission probabilities and counted the number of pathogenic bacteria that each individual has per cycle. As we can see in Table 3, when the bacterial pathogen transmission probability is 0.2, some individuals in a specific cycle (out of two million possibilities) became infected by three pathogenic bacteria. Therefore, we settled the bacterial pathogen transmission probability to be less than 0.2. Moreover, as shown in Table 4, the proportion of cycles that end without pathogens increases when pathogen transmission probability decreases. Therefore, we set this probability to 0.15 in the simulations.

### 3.2. Calibration of the Transmission Probability

As explained earlier, individuals take antibiotics when infected with pathogenic bacteria. However, antibiotics can select any resistant bacteria of the human microbiome, pathogenic or not. Consequently, antibiotic administration lowers the diversity of virulence genes, so it is essential to calibrate the probability of transmission of these genes so that they do not disappear from the network. These genes disappeared from the network when gene loss rate was higher than gene flow between individuals.

To better understand the impact of the gene transmission probability, we studied the simplest case, the one in which there is no fitness cost for harboring resistance genes (hence, resistance gene loss rate = 0), so only antibiotics can eliminate genes.

**Figure 3:**
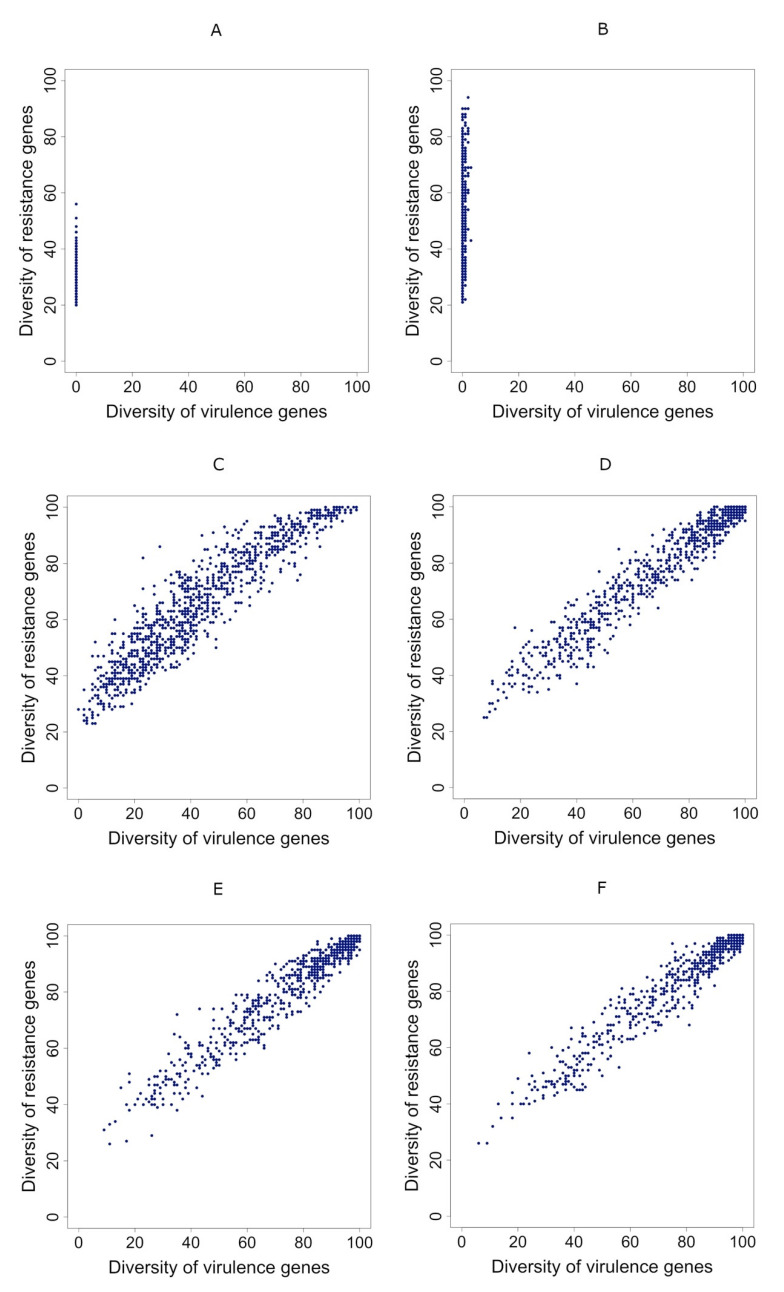
Effect of the gene transmission probability. (**A**–**F**): the relationship between the diversity of resistance genes (vertical axes) and the diversity of virulence genes (horizontal axes). Each dot represents the case of an individual metagenome. (**A**,**B**): disappearance of the diversity of virulence genes; (**C**,**D**): positive correlation between the diversity of resistance genes and the diversity of virulence genes; (**E**,**F**): positive correlation between the diversity of resistance genes and the diversity of virulence, with many individuals having a high diversity of the two gene types. Parameters as follows. In all cases, we set resistance genes loss rate = 0. In (**A**), when the gene transmission probability is low (0.0005), virulence genes disappeared from the network. In (**B**), gene transmission probability = 0.0025 (R = 0.309, slope = 11.00, *p*-value = 1.47 × 10^−23^). In (**C**), gene transmission probability = 0.005 (R = 0.934, slope = 0.798, *p*-value = ~0). In (**D**), gene transmission probability = 0.01 (R = 0.973, slope = 0.757, *p*-value = ~0). In (**E**), gene transmission probability = 0.015 (R = 0.972, slope = 0.754, *p*-value = ~0). In (**F**), gene transmission probability = 0.02 (R = 0.976, slope = 0.751, *p*-value = ~0).

As shown in Figure 3, when the gene transmission probability was below 0.005 (Figure 3A,B), virulence genes disappeared from the network. On the other hand, when the transmission probability of genes was above 0.01 (Figure 3E,F), several individuals had the maximum diversity of genes in their metagenome, which does not correspond to the observation by Escudeiro et al. (2019) (ref. [16]). Following these results, the gene transmission probability should be 0.005 or 0.01 (Figure 3C,D).

### 3.3. Correlation between Diversities Is Positive if Gene Transmission Probability Is Higher Than the Resistance Gene Loss Rate

**Figure 4:**
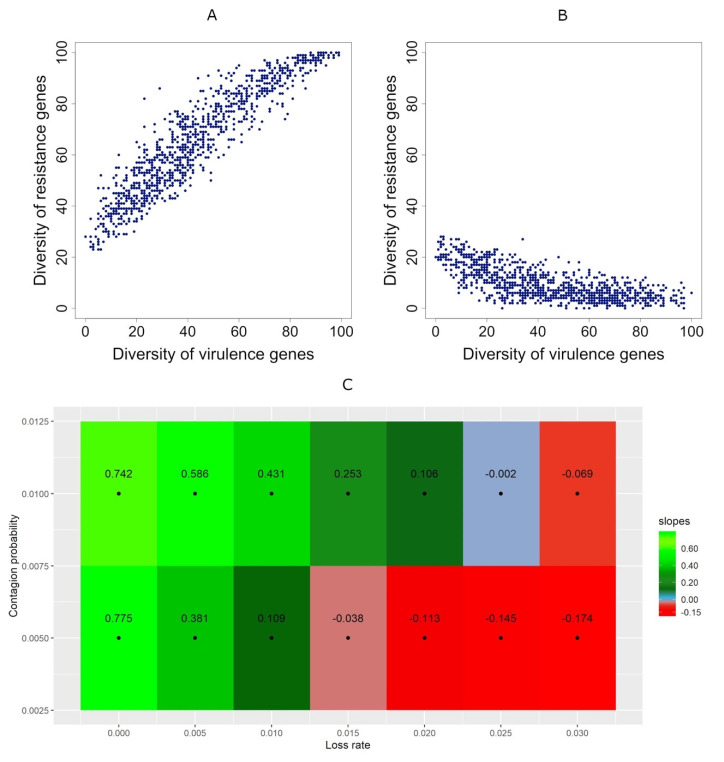
Effect of the relative values of the gene transmission probability and the resistance genes loss rate. (**A**,**B**): the relationship between the diversity of virulence genes (horizontal axes) and the diversity of resistance genes (vertical axes). Each dot represents the case of an individual metagenome. In both (**A**) and (**B**), the gene transmission probability = 0.005. (**A**): resistance genes loss rate = 0, which is lower than the gene transmission probability, resulting in a positive slope; (R = 0.929, slope = 0.775, *p*-value ~ 0). (**B**): resistance genes loss rate = 0.03, which higher than the gene transmission probability, resulting in a negative slope; (R = -0.682, slope = −0.174, *p*-value = 1.19 × 10^−137^). (**C**): Slope of the regression between the diversity of virulence and resistance genes according to the loss rate (horizontal axes) and the gene transmission probability (vertical axes). Green: positive slopes; Red: negative slopes; Blue: the slope is not significantly different from zero (*p*-value ≥ 1 × 10^−6^).

With the system already calibrated, we studied the correlation between virulence genes’ diversity and resistance genes’ diversity for various gene transmission probability and resistance gene loss rates. For that, we fixed all the other parameters (see Table 2). Figure 4 shows that, if the gene transmission probability is higher, the same, or only slightly lower than the loss rate, the correlation between the diversity of virulence genes and the diversity of resistance genes is positive (Suppl. Table S1, Figure 4).

### 3.4. Correlations Maintain Sign Even when People Take Antibiotics Randomly

In the preceding section, we concluded that the correlation between virulence and resistance gene diversities is positive if the genes’ transmission probability is higher than the resistance gene loss rate. However, such correlation could, at least in part, result from the spread of the pathogen that leads to antibiotic administration. Here, we evaluate what happens if individuals take antibiotics at random, not because they are coping with a bacterial infection. We chose these individuals randomly in each cycle. In the previous simulations where antibiotic intake was a consequence of the pathogen presence, there were 13 out of 1000 individuals, on average, taking antibiotics in each cycle. Thus, in this section, we considered that the probability that a random individual takes antibiotics is 0.013. At the end of simulations, we obtained the same correlations’ signs (either a positive or a negative correlation), when we compare the two situations, random antibiotic intake versus caused by pathogen spread through the network (compare Suppl. Table S1 and Figure 4C with Suppl. Table S2.1 and Suppl. Figure S1.1, respectively). In other words, whatever the reasons for taking antibiotics are, the correlation between diversities is positive if gene transmission probability is higher than the resistance gene loss rate.

### 3.5. Taking Antibiotics Is Crucial for a Positive Correlation between Virulence and Resistance Genes’ Diversity in Metagenomes

In the previous sections, we showed that, independently of the cause for antibiotic administration, the positive correlation between virulence and resistance genes’ diversity emerges if the gene transmission probability is higher than the loss rate. Here, we ask if taking antibiotics by people is crucial for this outcome.

If no one takes antibiotics, there is no counter-selective pressure on commensal bacteria encoding virulence genes. The result is that virulence genes’ diversity gets the maximum possible value in everyone’s metagenome in the community (in Suppl. Figure S2.1A,B,C, all the dots converge to the right). If the loss rate is null (if there is no fitness cost of resistance), all metagenomes accumulate every possible virulence and resistance gene families, so their diversity attains the maximum achievable value (in Suppl. Figure S2.1A, one can see that all the dots congregate to a single point at the top right corner). If the loss rate is low, there is some diversity of resistance genes in the population (in Suppl. Figure S2.1B, all the dots distribute in a vertical line on the right side). Finally, if the loss rate is high, more resistance genes are lost than those that accumulate through transmission, so all metagenomes lose all resistance genes (see Suppl. Figure S2.1C, where all the dots congregate to a single point at the bottom right corner).

### 3.6. Positive Correlations Are Robust under Changes in the Main Simulated System’s Properties

We have seen that the positive correlation between virulence and resistance genes’ diversity holds if the gene transmission probability is higher than the loss rate (Figure 4C). We then analyzed the robustness of this result. The following five subsections show the impact of changing some of the simulations’ variables, including network type. We studied the following variables: population size, the ratio between virulence genes and antibiotic resistance genes, the elimination probability under antibiotic intake, the proportion of the population harboring antibiotic-resistance genes in their metagenome, and the network type (regular, small-world, or random).

#### 3.6.1. Positive Correlations Are Robust under Changes in the Population Size

In most simulations, we assumed that the human population has just a thousand people due to computing power constraints. Therefore, it is essential to investigate whether population size impacts the correlations’ signs. We performed simulations with a population size of 3000 individuals, instead of 1000 individuals, for the 14 conditions shown in Figure 4C. Although there were significant differences between the slopes in three cases, we didn’t observe a change of the correlation’s sign from the cases where the population size was 1000 individuals (Suppl. Table S3.1 and Suppl. Figure S3.1). An increase in the population size leads to a rise in the number of intermediaries between two distant individuals. Therefore, for virulence genes and antibiotic resistance genes to be transferred between these two faraway individuals, more contacts are needed, and, consequently, more time is required to achieve a stable correlation.

#### 3.6.2. Positive Correlations Are Robust under Changes in the Ratios between Virulence and Antibiotic Resistance Genes Diversities

In all the other sections, we considered that virulence and resistance genes have the same total diversity. Here, we studied the effect of assuming that the diversity of virulence genes is different from that of resistance genes for the same 14 conditions of gene transmission probability and loss rate studied in the previous section. For that, we performed simulations similar to the previous ones, but with the following ratios between virulence and antibiotic resistance genes: 1:2, 1:4, 2:1, 4:1. Although there were significant differences between the slopes in 48 out of 56 cases, we did not detect any change in the correlation’s sign (Suppl. Tables S4.1 to S4.4 and Suppl. Figures S4.1 to S4.4).

#### 3.6.3. Positive Correlations Are Robust under Changes in the Gene Elimination Probability when People Take Antibiotics

When an individual takes an antibiotic, virulence genes and resistance genes are eliminated from the metagenome with a probability of 0.7 (except for resistance genes corresponding to the antibiotic used, which are selected, not eliminated). In this section, we analyzed the impact of using other elimination probabilities when an individual takes an antibiotic because the proportion of resistant cells in a bacterial community may change due to horizontal gene transfer. For that, we performed simulations similar to the previous ones, for the same 14 conditions of gene transmission probability and loss rate, but where the probability of eliminating genes under antibiotic intake is 0.3 and 0.5 for all gene types (instead of 0.7). In 19 out of 28 cases, the slopes were not significantly different from those obtained with a probability of 0.7 (Suppl. Tables S5.1 and S5.2). The slopes were different in the other nine cases, but the sign remained the same (that is, correlations are still positive or negative if they are already positive or negative, respectively, for the elimination probability of 0.7) (Suppl. Tables S5.1 and S5.2 and Suppl. Figures S5.1 and S5.2).

We also checked the impact of setting the probability of eliminating antibiotic resistance genes different from that of eliminating virulence genes. Although the slopes were significantly different in 51 out of 84 tested cases, the slopes’ sign remained the same (Suppl. Tables S6.1 to S6.6 and Suppl. Figures S6.1 to S6.6). Overall, these results show that the slope’s sign is robust under changes in the elimination probability.

#### 3.6.4. Positive Correlations Are Robust under Changes in the Initial Proportion of Metagenomes Containing Antibiotic-Resistance Genes

In the previous sections, we considered that every individual carries all the antibiotic resistance genes in two alternative states at the beginning of the simulation. The two states are: (i) either resistance genes were present at low copy numbers (hence being very unlikely to be transmitted to other people) or (ii) resistance genes are at high copy numbers caused by recent antibiotic exposure (thus with a high chance of being transmitted to other people). In this section, we study the effect of considering that, initially, only 10% of the metagenomes contain antibiotic-resistance genes. With this parameter changed, the simulations take more time to stabilize because 90% of the population receives resistance genes only through the transmission. We performed simulations similar to the ones shown in Figure 4 but with 5000 cycles. The final slopes are not significantly different from the case where all metagenomes initially contain antibiotic-resistance genes (Suppl. Table S7.1 and Suppl. Figure S7.1).

#### 3.6.5. Positive Correlations Are Robust under Changes in the Network Type

The simulations leading to Figure 4 were performed in a network with a rewiring probability of *p* = 0.5 (see Methods). We then performed similar simulations but in a regular (*p* = 0) and in random (*p* = 1) networks. This parameter did not change the correlation signs (see Suppl. Tables S8.1 and S8.2). However, the time needed (number of cycles) to reach a stable distribution was lower for higher values of p, this being explained by the low characteristic path length and the low clustering coefficient when p is high (random graph) (Suppl. Figure S8.3).

In conclusion, the correlation between virulence and resistance genes’ diversity is positive if the gene transmission probability is higher than the loss rate of antibiotic-resistance genes, even when we change following variables: the cause for antibiotic administration (randomly or due to pathogen spread); population size; the ratio between the total availability of virulence antibiotic resistance genes in the community; the elimination probability under antibiotic intake; the proportion of the population harboring antibiotic-resistance genes in their metagenome; and the network type (regular network, small-world network, and random network).

## 4. Discussion

Antibiotics affect hundreds of commensal and mutualist bacterial strains and species, even if their administration aims at targeting bacterial pathogens. Moreover, healthy animals often take antibiotics, given the properties of these drugs as growth-promoters. With these two processes, antibiotic-sensitive bacteria are counter-selected, raising the frequency of antibiotic resistance cells in metagenomes. Meanwhile, metagenomes from sick and healthy people harbor virulence genes. This paper aimed to understand why there is a positive correlation between the diversity of virulence and antibiotic-resistance genes among human populations’ microbiomes [16]. For that, we developed a computational model tailored to address the relationship between antibiotic-resistance genes and virulence genes.

Since this is the first study ever performed to address this issue, we chose to keep the simulations as general as possible. However, it can be adapted to generate simulations adjusted to specific antibiotics or other health scenarios in future studies.

Our simulations’ main result is that a positive correlation emerges if the gene transmission probability is higher than the loss rate of antibiotic-resistance genes. We can understand this result in the following way.

Bacterial pathogens, antibiotic resistance genes and virulence genes flow between human individuals’ microbiomes in a network of contacts. In the absence of bacterial pathogens, people do not take antibiotics in that particular cycle; in these people, the diversity of virulence genes increase through transmission from their contacts in the network. However, for the case of antibiotic resistance genes, two opposing forces play a role in the microbiomes of people not taking antibiotics: (i) transmission from other people in the network makes the diversity of resistance genes increase, whereas (ii) gene loss, due to fitness cost imposed by resistance determinants, decreases it. At the end of a cycle, the diversity of resistance genes increases exclusively if the effect of transmission is higher than that of gene loss. The gene loss is just the consequence of the fitness cost imposed by resistance determinants (e.g., chromosomal mutations or mobile genetic elements) in competition with susceptible cells. However, the transmission effect has two main contributors: the transmission probability and the number of connections, which depends on the network type and varies from person to person in non-regular networks. Altogether, Figure 4C and the corresponding figures in Supplementary File (Suppl. Figures S3.1, S4.1–S4.4, S5.1, S5.2, S6.1–S6.6, S7.1, S8.1, and S8.2) show that, if the transmission rate is higher than the loss rate of antibiotic-resistance genes, a positive correlation emerges between the diversity of antibiotic resistance genes and virulence genes.

At first, one might expect to see a negative correlation whenever the transmission probability is lower than the loss rate. Yet, we have seen that, when the transmission probability is only slightly lower than the loss rate, for example, if the transmission probability is 0.005 and the loss rate is 0.01, the correlation is still positive (Figure 3C and Figure 4A, Suppl. Table S1). The reason for these counter-intuitive cases is that, in each cycle, one individual contacts with four other individuals, and during each of these contacts, they share bacteria from its microbiomes. In turn, each individual can only be medicated with antibiotics once (at the end of a cycle). That implies that the rate of loss of resistance genes applies only once in a cycle. Therefore, the impact of the transmission rate is higher than the loss rate of resistance genes.

We assumed that resistance determinants are already present in low amounts in all metagenomes because they are a part of the natural bacterial lifestyle, and human beings have used massive quantities of antibiotics since the 1940s [42,43]. What is the impact of this assumption? As shown in Suppl. Table S7.1, if we assumed that, initially, only 10% of the metagenomes contain antibiotic-resistance genes, the only difference is that more cycles are needed to stabilize the correlation. The final correlations between the diversity of resistance genes and the diversity of virulence genes are the same as in the default case. Moreover, we have seen in Section 3.4 that, even if antibiotic consumption in the population is random, to simulate the improper use of antibiotics (i.e., unrelated to infection by the pathogen), the correlation sign does not change. This result indicates that the positive correlations between the diversity of resistance genes and the diversity of virulence genes may not result from antibiotic misuse.

The transmission probability between people and the loss rate of antibiotic-resistance genes are the two critical parameters of our main result, so it is relevant to know them. The interest and attention drawn to the Human microbiome have enormously increased in recent years, yet we still do not know much about the dynamics of the flow of non-housekeeping genes in shared microbiomes.

Sarowska and colleagues recently reviewed the fate of the extraintestinal pathogenic *Escherichia coli* (ExPEC), which are facultative pathogens of the normal human intestinal microbiome. ExPEC pathogenicity relies on many virulence genes, and pathogenicity islands, or mobile genetic elements (such as plasmids) encoding some of them. One of the authors’ conclusions is precisely the difficulty in assigning ExPEC transmission to people due to the delay between ExPEC colonization and infection: ExPEC cells can live in human intestines for months or even years before starting an infection [44]. The same problem applies to the transmission rate of antibiotic-resistance genes: there is very little data on transmission rates between people [43].

We observed that what is essential to understand the positive correlation between resistance and virulence genes diversity are not the exact values for the transmission rates but the relationship between the transmission and loss rates. Therefore, we now discuss how much is the loss rate of resistance determinants in human metagenomes. Several longitudinal studies have shown that antibiotic-resistance genes often remain tens of days, sometimes months, in human gut microbiomes [45,46,47,48]. While still harboring resistance genes, people most probably contact several other people. Yet, the relationship between transmission and loss rates is still unclear.

As explained in the methods section, the loss of antibiotic resistance results from the fitness cost of resistance determinants on bacterial cells (compared to otherwise isogenic susceptible cells). Several studies have shown that resistance determinants, here broadly comprising resistance mutations and resistance genes encoded in the chromosome or plasmids, frequently impose a fitness cost on their hosts (giving the sensitive strains a growth advantage) [49]. However, several mechanisms decrease or even eliminate it. First, compensatory mutations, which mask the deleterious effects of resistance mutations, have been observed in several studies [50,51,52,53,54]. Second, resistance mutations can be beneficial in specific resistance genetic backgrounds, e.g., through epistatic interactions with other chromosomal mutations [25]. Third, while resistance plasmids often impose a fitness cost to their hosts, it has also been observed that plasmids and/or cells need just a few tens or hundreds of bacterial generations to adapt to each other [55,56,57,58,59,60]. Fourth, plasmids sometimes increase the fitness of bacteria that already harbor a resistance mutation [26]; likewise, some resistance mutations increase the fitness of plasmid-bearing cells [26]. The same may happen with two plasmids: one of them compensating for the fitness-cost of the other [26,61]. Fifth, plasmids may interact with other plasmids to facilitate their transfer [62,63,64,65]. Sixth, a few studies suggested that plasmids appear costly because their fitness effect is often measured a long time after its isolation from nature [65,66]. Seventh, transferable plasmids may have adaptive value to their hosts as promoters of bacterial biofilms formation [67,68,69]. Biofilms often confer protection to bacteria against harmful agents, including antibiotics.

Together, these factors suggest that the fitness cost of resistance determinants is often very low or null, allowing the permanence of resistance determinants in microbiomes for long periods. Some resistance determinants may even be beneficial in the absence of antibiotics [70]. This stability of resistance determinants implies that their loss rate, the probability that a metagenome loses a particular resistance gene or mutation, is undoubtedly lower than the transmission probability. Therefore, antibiotic consumption and transmission between people lead to a positive correlation between the diversity of resistance genes and virulence genes.

We have shown that the network structure has a low influence on the final correlation between virulence and resistance genes’ diversity. However, there is a difference in the total time needed to achieve the final distribution, being much lower when the structure is random or in a small-world regime. The cause of such faster convergence is the small characteristic path length (both of random and small-world network types), facilitating transmission between people. This result agrees with previous studies showing, for example, that the shorter path lengths in small-world networks increase the efficiency of natural selection [71]. It is natural to consider that the structure of microbial populations is related to the structure of contacts of their hosts. In humans, the network of friendships in a high school [72,73] and sexual contacts in Sweden [74] have a small-world property (also see Reference [75]).

We already knew that people with a higher diversity of resistance genes in their gut metagenomes have a higher diversity of virulence genes [16] and that the presence of both types of adaptive accessory traits in a microbiome may potentiate the appearance of plasmids or bacteria encoding virulence and resistance genes simultaneously and prompting their spreading into different bacteria [16]. However, the data shown here has further worrying health implications. While people with lower diversity of virulence genes also tend to have a lower diversity of resistance genes, the simulations presented here show that these people are precisely those that have recently taken antibiotics. Although the overall diversity of resistance genes is minimal in these people, they probably have a high concentration of certain resistance genes. These genes can then be transferred horizontally to cells encoding virulence genes.

## 5. Concluding Remarks

In this study, we tested the hypothesis that the correlation between the diversity of antibiotic resistance genes and virulence encoding genes is due to the flow of bacteria and genes between individuals within a human contact network, using simulation-based experiments with a statistically significant association between cause and effect.

Bacterial transmission and antibiotic use are chief to explain the positive correlation between antibiotic resistance gene diversity and virulence gene diversity across human metagenomes. This result is robust and general because we made very few assumptions and remains valid under changes of several relevant variables (data and respective *p*-values in Suppl. Tables S2.1 to S8.2).

## Figures and Tables

**Table 1:** Pseudocode of the program *.

Process	Pseudo Code
Gene transfer	For each connection between two individuals do (for each individual of the connection do (get the genes present in each individual metagenome; transmit genes to the other individual of the connection according to the gene transmission probability)).
Transfer of bacterial pathogens	For each connection between two individuals do (for each individual of the connection do (get the pathogenic species present in each individual; transmit pathogen to the other individual of the connection according to the bacterial pathogen transmission probability)).
Screening of individuals	For each individual do (check if the individual has a pathogenic bacteria).
Antibiotic effect	Choose an antibiotic randomly. Select all resistance genes associated with the chosen antibiotic. Eliminate resistance genes not associated with the chosen antibiotic according to the probability of eliminating genes under antibiotic intake. Eliminate virulence genes according to the probability of eliminating genes under antibiotic intake.
Loss rate of resistance genes under antibiotic consumption	Eliminate resistance genes not associated with the chosen antibiotic due to fitness cost according to the loss rate probability.
Loss rate of resistance genes without antibiotic consumption	Eliminate resistance genes according to the loss rate probability.
Immigration of bacterial pathogen into the network	For each bacterial species do (select a random individual; insert the bacterial pathogen in the individual).

* Pseudocode of the program sketched in Figure 2. The program code was implemented in the Python programming language.

**Table 2:** Parameters and default values used in simulations.

Parameters	Default Values	Other Values
Rewiring connectivity probability *p*	0.5	0 or 1
Number of individuals	1000	3000
Number of virulence genes	100	200, 400
Number of resistance genes	100	200, 400
Number of pathogenic bacterial species	5	NA
Number of antibiotics	5	NA
Gene transmission probability	0.005, 0.01	0.0005, 0.0025, 0.015, 0.02
Bacterial pathogen transmission probability	0.15	0.05, 0.1, 0.2, 0.25
Probability of eliminating genes under antibiotic intake	0.7	0.3, 0.5
The loss rate of resistance genes	0, 0.005, 0.01, 0.015, 0.02, 0.025, 0.03	NA

**Table 3:** Number of pathogenic species according to the bacterial pathogen transmission probability.

	Number of Pathogenic Species (in 2,000,000 Possibilities)					
Bacterial Pathogen Transmission Probability	0	1	2	3	4	5
0.05	1,987,473	12,496	31	0	0	0
0.1	1,982,852	17,094	54	0	0	0
0.15	1,973,053	26,763	184	0	0	0
0.2	1,940,458	58,759	779	4	0	0
0.25	104,967	262,575	527,204	705,479	399,253	522

**Table 4:** Simultaneous extinction of all pathogenic bacterial species according to the bacterial pathogen transmission probability.

Bacterial Pathogen Transmission Probability	Number of Times that All Pathogenic Bacterial Species Disappeared in a Cycle (in 2000 Possibilities)
0.05	570
0.1	70
0.15	2

## Data Availability

All data is freely available in Supplementary Materials and source code from GitHub.

## Supplementary Materials

The following are available online at https://www.mdpi.com/article/10.3390/antibiotics10050605/s1, Table S1: Parameters used for Figure 4C and the results of the simulations, Table S2.1: The impact of considering random consumption of antibiotics on the correlation between virulence and resistance genes, Figure S1.1: Effect of considering random consumption of antibiotics, Figure S2.1: Effect of considering no consumption of antibiotics, Table S3.1: The impact of considering 3000 individuals on the correlation between virulence and resistance genes, Figure S3.1: Effect of considering bigger populations (3000 individuals), Table S4.1: The impact of considering a ratio of 1 virulence gene for 2 resistance genes on the correlation between virulence and resistance genes, Figure S4.1: Effect of considering a ratio of 1 virulence gene for 2 resistance genes, Table S4.2: The impact of considering a ratio of 1 virulence gene for 4 resistance genes on the correlation between virulence and resistance genes, Figure S4.2: Effect of considering a ratio of 1 virulence gene for 4 resistance genes, Figure S4.3: Effect of considering a ratio of 2 virulence genes for 1 resistance gene, Table S4.4: The impact of considering a ratio of 4 virulence genes for 1 resistance gene on the correlation between virulence and resistance genes, Figure S4.4: Effect of considering a ratio of 4 virulence genes for 1 resistance gene, Table S5.1: The impact of considering a probability of eliminating genes under antibiotic intake of 30% on the correlation between virulence and resistance genes, Figure S5.1: Effect of considering a probability of eliminating genes under antibiotic intake of 30%, Table S5.2: The impact of considering a probability of eliminating genes under antibiotic intake of 50% on the correlation between virulence and resistance genes, Figure S5.2: Effect of considering a probability of eliminating genes under antibiotic intake of 50%, Table S6.1: The impact of considering a probability of eliminating virulence genes under antibiotic intake of 30% and a probability of eliminating resistance genes under antibiotic intake of 50% on the correlation between virulence and resistance genes, Figure S6.1: Effect of considering a probability of eliminating virulence genes under antibiotic intake of 30% and a probability of eliminating resistance genes under antibiotic intake of 50%, Table S6.2: The impact of considering a probability of eliminating virulence genes under antibiotic intake of 30% and a probability of eliminating resistance genes under antibiotic intake of 70% on the correlation between virulence and resistance genes, Figure S6.2: Effect of considering a probability of eliminating virulence genes under antibiotic intake of 30% and a probability of eliminating resistance genes under antibiotic intake of 70%, Table S6.3: The impact of considering a probability of eliminating virulence genes under antibiotic intake of 50% and a probability of eliminating resistance genes under antibiotic intake of 30% on the correlation between virulence and resistance genes, Figure S6.3: Effect of considering a probability of eliminating virulence genes under antibiotic intake of 50% and a probability of eliminating resistance genes under antibiotic intake of 30%, Table S6.4: The impact of considering a probability of eliminating virulence genes under antibiotic intake of 50% and a probability of eliminating resistance genes under antibiotic intake of 70% on the correlation between virulence and resistance genes, Figure S6.4: Effect of considering a probability of eliminating virulence genes under antibiotic intake of 50% and a probability of eliminating resistance genes under antibiotic intake of 70%, Table S6.5: The impact of considering a probability of eliminating virulence genes under antibiotic intake of 70% and a probability of eliminating resistance genes under antibiotic intake of 30% on the correlation between virulence and resistance genes, Figure S6.5: Effect of considering a probability of eliminating virulence genes under antibiotic intake of 70% and a probability of eliminating resistance genes under antibiotic intake of 30%, Table S6.6: The impact of considering a probability of eliminating virulence genes under antibiotic intake of 70% and a probability of eliminating resistance genes under antibiotic intake of 50% on the correlation between virulence and resistance genes, Figure S6.6: Effect of considering a probability of eliminating virulence genes under antibiotic intake of 70% and a probability of eliminating resistance genes under antibiotic intake of 50%, Table S7.1: The impact of considering that, initially, only 10% of metagenomes contain antibiotic resistance genes on the correlation between virulence and resistance genes, Figure S7.1: Effect of considering that, initially, only 10% of metagenomes contain antibiotic resistance genes, Table S8.1: The impact of considering a random network (*p* = 1) on the correlation between virulence and resistance genes, Figure S8.1: Effect of considering a random network (*p* = 1), Table S8.2: The impact of considering a regular network (*p* = 0) on the correlation between virulence and resistance genes, Figure S8.2: Effect of considering a regular network (*p* = 0), Figure S8.3: Effect of the networks.
