# Supplementary material for: The Perfect Condition for the Rising of Superbugs: Person-to-Person Contact and Antibiotic Use Are the Key Factors Responsible for the Positive Correlation between Antibiotic Resistance Gene Diversity and Virulence Gene Diversity in Human Metagenomes
Source: Antibiotics (Basel). 2021 May 20;10(5):605. doi: 10.3390/antibiotics10050605 (PMC8161104; doi:10.3390/antibiotics10050605)
Supplement: Supplementary file 1 [file antibiotics-10-00605-s001.zip › antibiotics-1197802-supplementary.pdf]

Supplementary Information of the manuscript:

The perfect condition for the rising of superbugs: person-to-person contact and antibiotic use are the key factors responsible for the positive correlation between antibiotic resistance gene diversity and virulence gene diversity in human metagenomes

Célia P. F. Domingues[1, 2†], João S. Rebelo[1†], Joël Pothier[3], Francisca Monteiro[1], Teresa Nogueira[1, 2*], and Francisco Dionisio[1*]

[1] cE3c – Centre for Ecology, Evolution and Environmental Changes, Faculdade de Ciências, Universidade de Lisboa, 1749-016 Lisboa, Portugal
[2] INIAV - National Institute for Agrarian and Veterinary Research, Bacteriology and Mycology Laboratory, 2780 -157 Oeiras, Portugal
[3] Atelier de Bioinformatique, ISYEB, UMR 7205 CNRS MNHN UPMC EPHE, Muséum National d'Histoire Naturelle, CP 50, 45 rue Buffon F-75005 Paris, France

† These authors have contributed equally to this work

***Correspondence:**
Francisco Dionisio dionisio@fc.ul.pt
and Teresa Nogueira: teresa.nogueira@iniav.pt

Keywords: antibiotic resistance; virulence; microbiome; metagenomics; human gut; computer simulation

1- Results of Fig. 4C

Table S1. Parameters used for Fig. 4C and the results of the simulations.

Contagion probability (%)	Resistance genes loss rate (%)	Correlation (r) between resistance genes and virulence genes	Slope of the regression between resistance genes and virulence genes	P-value of the slope
**0.5**	0	0.929	0.775	0
	0.5	0.825	0.381	$5.28x10^{-250}$
	1	0.414	0.109	$1.24x10^{-42}$
	1.5	-0.17	-0.038	$6.76x10^{-8}$
	2	-0.472	-0.113	$1.29x10^{-56}$
	2.5	-0.586	-0.145	$4.10x10^{-93}$
	3	-0.682	-0.174	$1.19x10^{-137}$
**1**	0	0.966	0.742	0
	0.5	0.94	0.586	0
	1	0.848	0.431	$8.35x10^{-277}$
	1.5	0.671	0.253	$8.77x10^{-132}$
	2	0.362	0.106	$2.95x10^{-32}$
	2.5	-0.01	-0.002	$7.47x10^{-1}$
	3	-0.319	-0.069	$3.69x10^{-25}$

2 – Correlations maintain sign even when people take antibiotics randomly

Table S2.1. The impact of considering random consumption of antibiotics on the correlation between virulence and resistance genes.

Contagion probability (%)	Resistance genes loss rate (%)	Slope considering random antibiotic consumption	Slope considering antibiotic consumption due to pathogenic bacteria	P-value of T-test for differences of slopes	Change in the slope sign?
**0.5**	0	0.762	0.775	$3.41x10^{-1}$	No
	0.5	0.423	0.381	$2.31x10^{-4}$	No
	1	0.173	0.109	$1.75x10^{-8}$	No
	1.5	-0.009	-0.038	$3.55x10^{-3}$	No
	2	-0.082	-0.113	$1.50x10^{-3}$	No
	2.5	-0.147	-0.145	$8.57x10^{-1}$	No
	3	-0.149	-0.174	$3.06x10^{-3}$	No
**1**	0	0.742	0.742	$9.82x10^{-1}$	No
	0.5	0.588	0.586	$8.97x10^{-1}$	No
	1	0.440	0.431	$4.88x10^{-1}$	No
	1.5	0.282	0.253	$2.93x10^{-2}$	No
	2	0.120	0.106	$2.68x10^{-1}$	No
	2.5	0.038	-0.002	$4.54x10^{-4}$	Yes
	3	-0.036	-0.069	$8.98x10^{-4}$	No

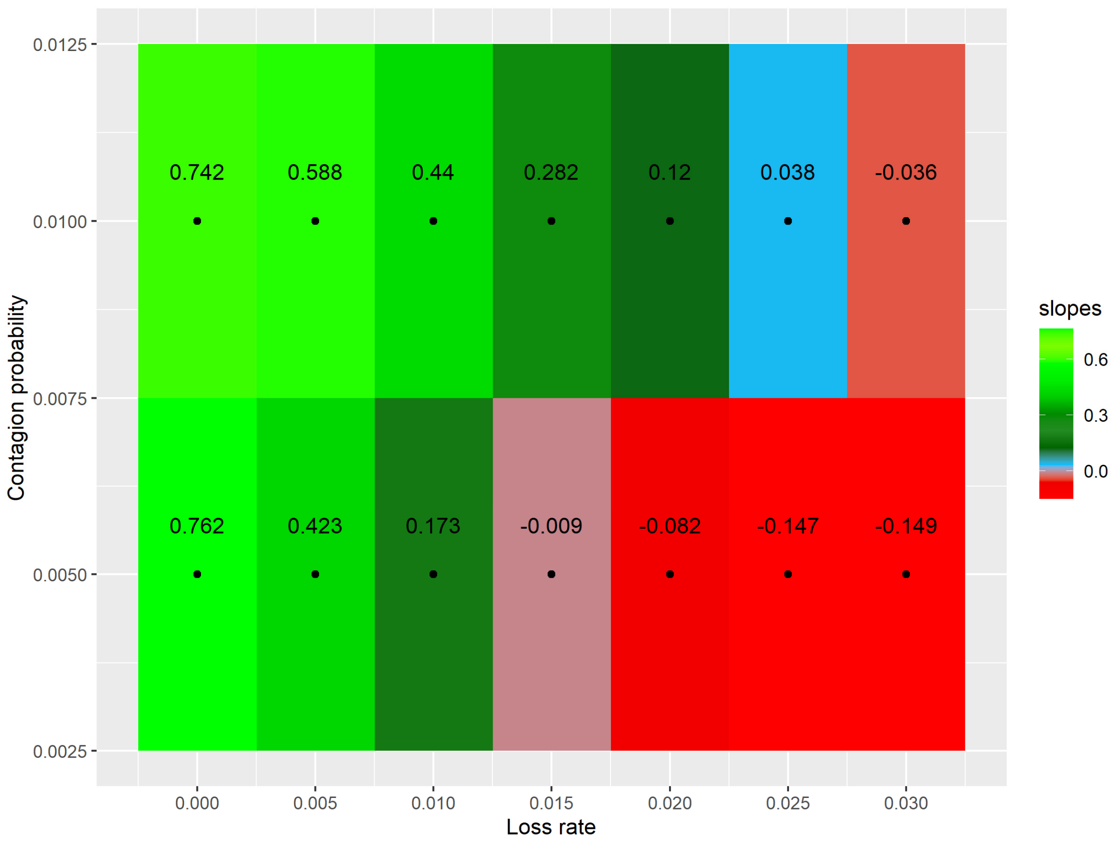

Figure S1.1. Effect of considering random consumption of antibiotics. Slope of the regression between the diversity of virulence genes and resistance genes according to the contagion probability (vertical axis) and the loss rate (horizontal axis). Green: positive slopes; and red: negative slopes.

3 – Taking antibiotics is crucial for a positive correlation between virulence and resistance genes' diversity

A) Resistance genes loss rate = 0

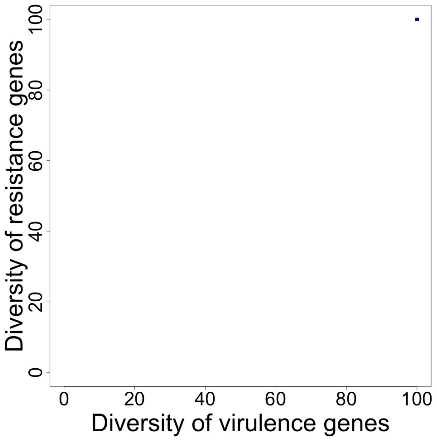

B) Resistance genes loss rate = 0.005

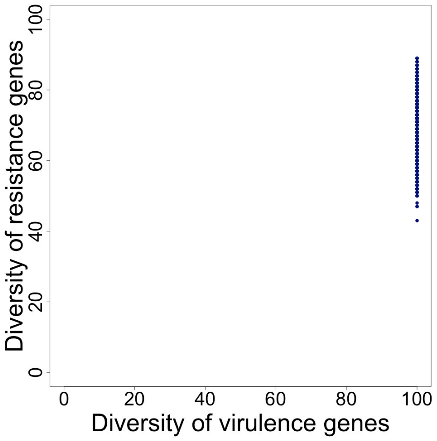

C) Resistance genes loss rate = 0.03

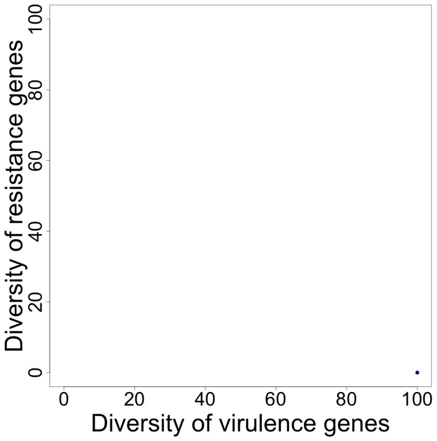

Figure S2.1. Effect of considering no consumption of antibiotics. A to C: the relationship between the diversity of resistance genes (vertical axes) and the diversity of virulence genes (horizontal axes). Each dot represents the case of an individual metagenome. A: accumulation of the diversity of virulence genes and resistance genes. B: . C: disappearence of the diversity of resistance genes; Parameters as follows. In A, B and C the gene contagion probability = 0.005.

4 - Population size has no impact on the correlation's sign

Table S3.1. The impact of considering 3000 individuals on the correlation between virulence and resistance genes.

Contagion probability (%)	Resistance genes loss rate (%)	Slope considering 3000 individuals	Slope considering 1000 individuals	P-value of T-test for differences of slopes	Change in the slope sign?
**0.5**	0	0.757	0.775	8.93×10^{-2}	No
	0.5	0.453	0.381	4.19×10^{-13}	No
	1	0.137	0.109	4.83×10^{-3}	No
	1.5	-0.078	-0.038	1.24×10^{-6}	No
	2	-0.147	-0.113	3.13×10^{-6}	No
	2.5	-0.166	-0.145	1.97×10^{-3}	No
	3	-0.160	-0.174	2.91×10^{-2}	No
**1**	0	0.769	0.742	1.03×10^{-4}	No
	0.5	0.640	0.586	2.24×10^{-9}	No
	1	0.484	0.431	1.12×10^{-5}	No
	1.5	0.326	0.253	2.55×10^{-8}	No
	2	0.152	0.106	2.07×10^{-4}	No
	2.5	0.007	-0.002	3.53×10^{-1}	Yes
	3	-0.106	-0.069	1.35×10^{-5}	No

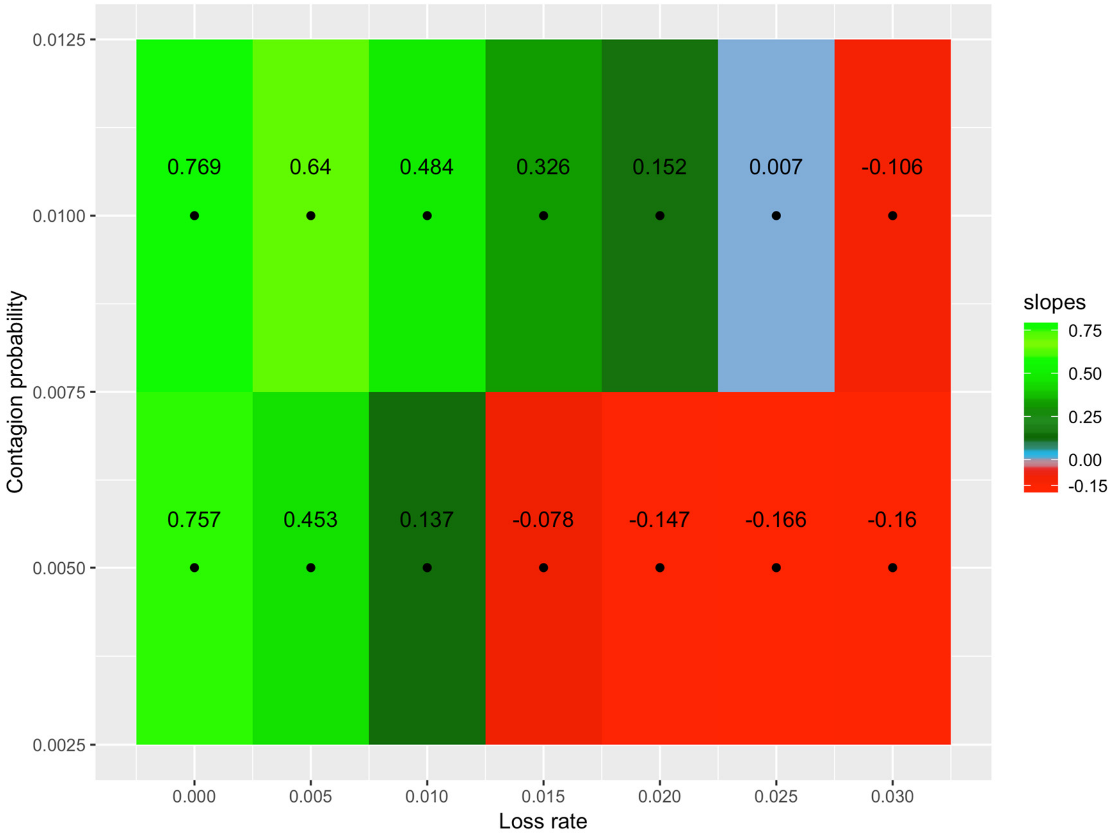

Figure S3.1. Effect of considering bigger populations (3000 individuals). Slope of the regression between the diversity of virulence genes and resistance genes according to the contagion probability (vertical axis) and the loss rate (horizontal axis). Green: positive slopes; and red: negative slopes.

5 - The ratios between virulence and antibiotic resistance genes diversities have no impact on correlation's sign

Table S4.1. The impact of considering a ratio of 1 virulence gene for 2 resistance genes on the correlation between virulence and resistance genes.

Contagion probability (%)	Resistance genes loss rate (%)	Slope considering a ratio of 1:2	Slope considering a ratio of 1:1	P-value of T-test for differences of slopes	Change in the slope sign?
**0.5**	0	1.591	0.775	$1.015073 \times 10^{-285}$	No
	0.5	0.836	0.381	$5.669288 \times 10^{-138}$	No
	1	0.235	0.109	1.553558×10^{-15}	No
	1.5	-0.094	-0.038	1.068848×10^{-4}	No
	2	-0.213	-0.113	5.657481×10^{-13}	No
	2.5	-0.291	-0.145	3.064856×10^{-29}	No
	3	-0.317	-0.174	1.175099×10^{-28}	No
**1**	0	1.515	0.742	0	No
	0.5	1.177	0.586	6.56×10^{-288}	No
	1	0.862	0.431	6.60×10^{-123}	No
	1.5	0.548	0.253	6.89×10^{-51}	No
	2	0.257	0.106	3.33×10^{-17}	No
	2.5	0.039	-0.002	6.35×10^{-3}	Yes
	3	-0.128	-0.069	4.25×10^{-5}	No

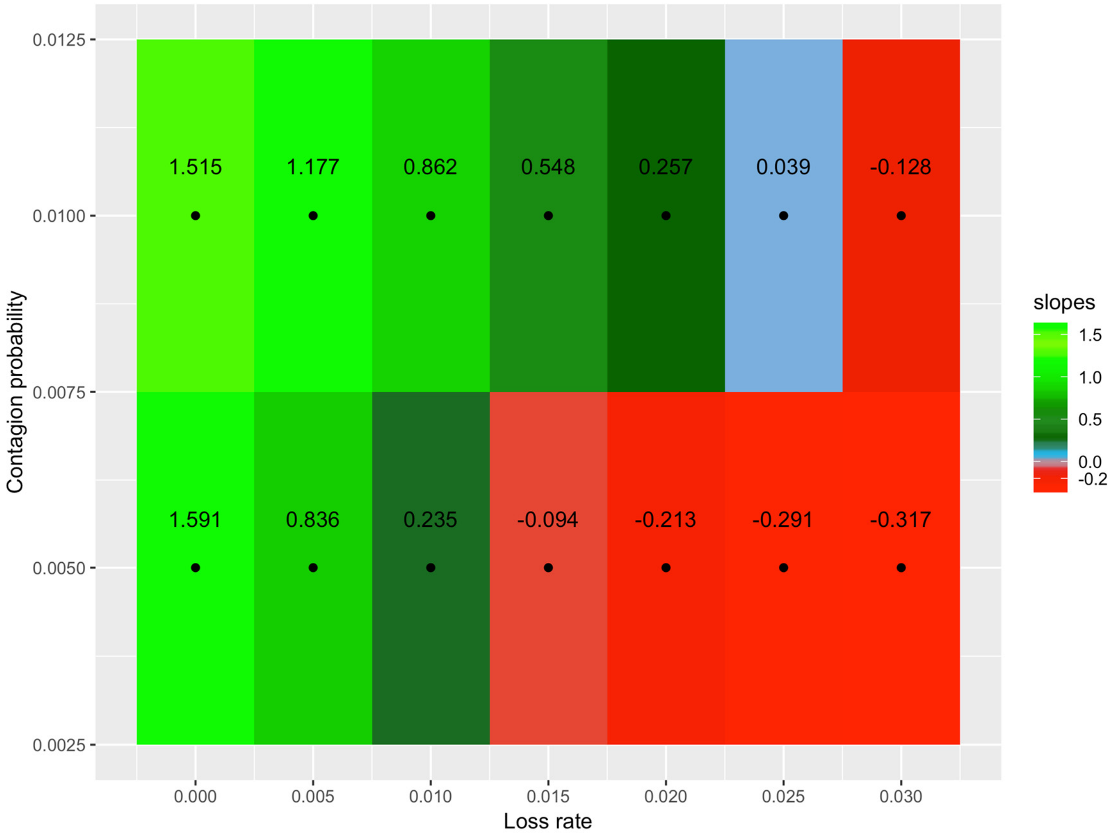

Figure S4.1. Effect of considering a ratio of 1 virulence gene for 2 resistance genes. Slope of the regression between the diversity of virulence genes and resistance genes according to the contagion probability (vertical axis) and the loss rate (horizontal axis). Green: positive slopes; and red: negative slopes.

Table S4.2. The impact of considering a ratio of 1 virulence gene for 4 resistance genes on the correlation between virulence and resistance genes.

Contagion probability (%)	Resistance genes loss rate (%)	Slope considering a ratio of 1:4	Slope considering a ratio of 1:1	P-value of T-test for differences of slopes	Change in the slope sign?
**0.5**	0	3.115	0.775	0	No
	0.5	1.592	0.381	3.68×10^{-275}	No
	1	0.530	0.109	3.36×10^{-50}	No
	1.5	-0.165	-0.038	6.52×10^{-8}	No
	2	-0.484	-0.113	7.66×10^{-56}	No
	2.5	-0.571	-0.145	1.13×10^{-76}	No
	3	-0.665	-0.174	2.05×10^{-92}	No
**1**	0	3.012	0.742	0	No
	0.5	2.376	0.586	0	No
	1	1.684	0.431	1.25×10^{-269}	No
	1.5	1.086	0.253	9.29×10^{-128}	No
	2	0.454	0.106	2.54×10^{-27}	No
	2.5	0.088	-0.002	1.02×10^{-3}	Yes
	3	-0.255	-0.069	4.10×10^{-18}	No

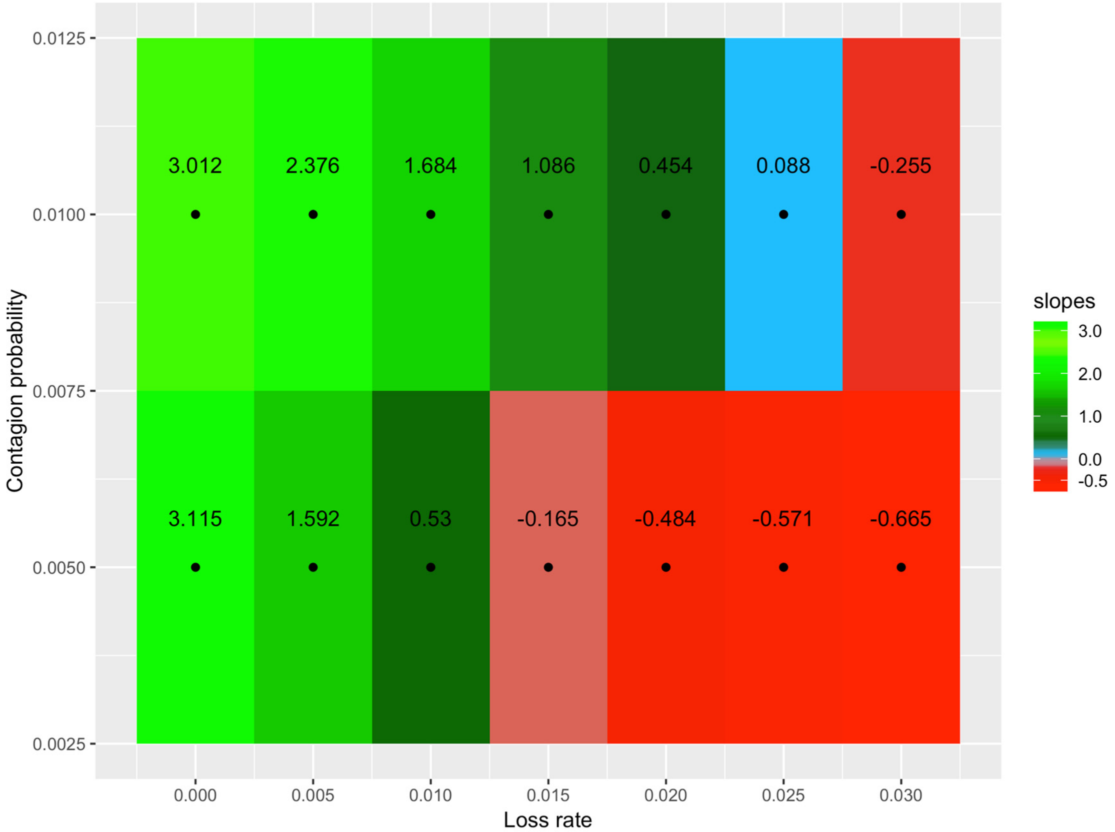

Figure S4.2. Effect of considering a ratio of 1 virulence gene for 4 resistance genes. Slope of the regression between the diversity of virulence genes and resistance genes according to the contagion probability (vertical axis) and the loss rate (horizontal axis). Green: positive slopes; and red: negative slopes.

Table S4.3. The impact of considering a ratio of 2 virulence genes for 1 resistance gene on the correlation between virulence and resistance genes.

Contagion probability (%)	Resistance genes loss rate (%)	Slope considering a ratio of 2:1	Slope considering a ratio of 1:1	P-value of T-test for differences of slopes	Change in the slope sign?
**0.5**	0	0.402	0.775	$2.05x10^{-208}$	No
	0.5	0.214	0.381	$7.16x10^{-70}$	No
	1	0.061	0.109	$9.67x10^{-9}$	No
	1.5	-0.020	-0.038	$2.08x10^{-2}$	No
	2	-0.055	-0.113	$1.43x10^{-14}$	No
	2.5	-0.070	-0.145	$1.61x10^{-25}$	No
	3	-0.084	-0.174	$2.24x10^{-40}$	No
**1**	0	0.374	0.742	0	No
	0.5	0.298	0.586	$1.19x10^{-239}$	No
	1	0.213	0.431	$4.32x10^{-104}$	No
	1.5	0.131	0.253	$2.02x10^{-33}$	No
	2	0.058	0.106	$4.21x10^{-7}$	No
	2.5	0.011	-0.002	$1.16x10^{-1}$	Yes
	3	-0.031	-0.069	$2.17x10^{-7}$	No

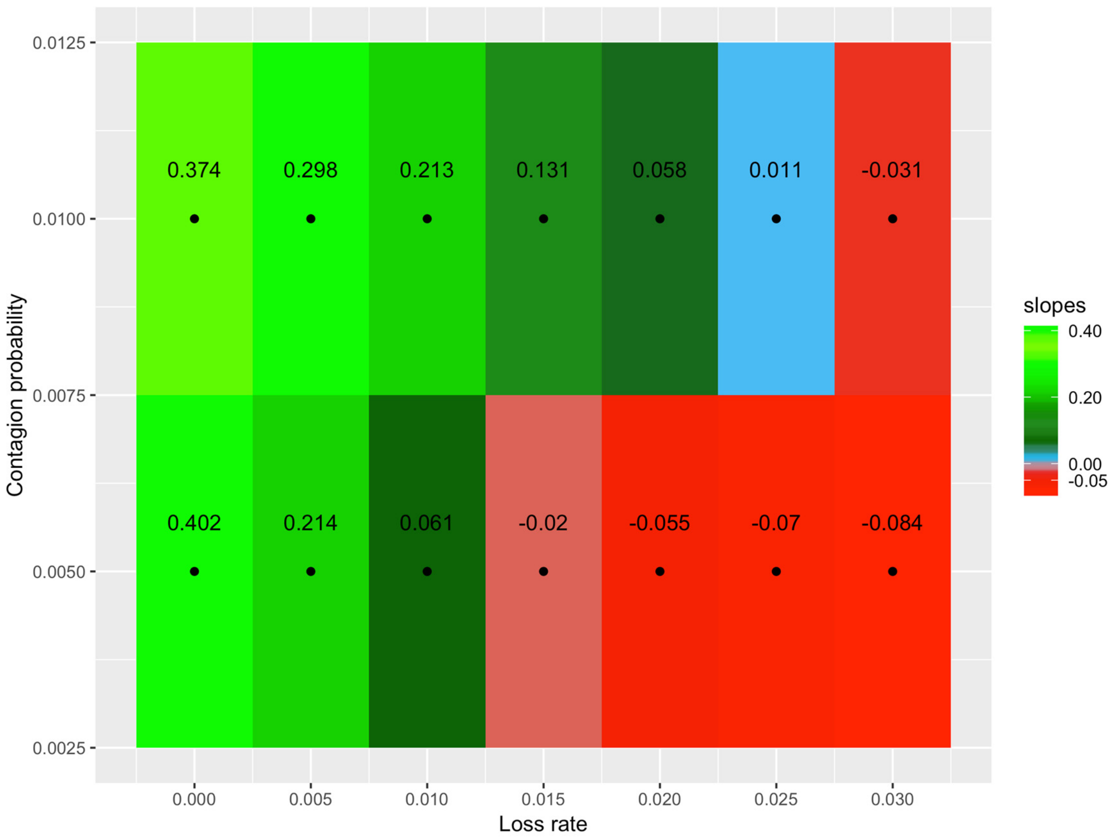

Figure S4.3. Effect of considering a ratio of 2 virulence genes for 1 resistance gene. Slope of the regression between the diversity of virulence genes and resistance genes according to the contagion probability (vertical axis) and the loss rate (horizontal axis). Green: positive slopes; and red: negative slopes.

Table S4.4. The impact of considering a ratio of 4 virulence genes for 1 resistance gene on the correlation between virulence and resistance genes.

Contagion probability (%)	Resistance genes loss rate (%)	Slope considering a ratio of 4:1	Slope considering a ratio of 1:1	P-value of T-test for differences of slopes	Change in the slope sign?
**0.5**	0	0.196	0.775	0	No
	0.5	0.099	0.381	5.03×10^{-192}	No
	1	0.033	0.109	4.18×10^{-22}	No
	1.5	-0.008	-0.038	3.17×10^{-5}	No
	2	-0.026	-0.113	1.03×10^{-35}	No
	2.5	-0.041	-0.145	4.99×10^{-54}	No
	3	-0.042	-0.174	1.52×10^{-93}	No
**1**	0	0.192	0.742	0	No
	0.5	0.146	0.586	0	No
	1	0.110	0.431	3.09×10^{-224}	No
	1.5	0.071	0.253	8.51×10^{-80}	No
	2	0.030	0.106	4.78×10^{-17}	No
	2.5	-0.001	-0.002	8.26×10^{-1}	No
	3	-0.014	-0.069	5.57×10^{-16}	No

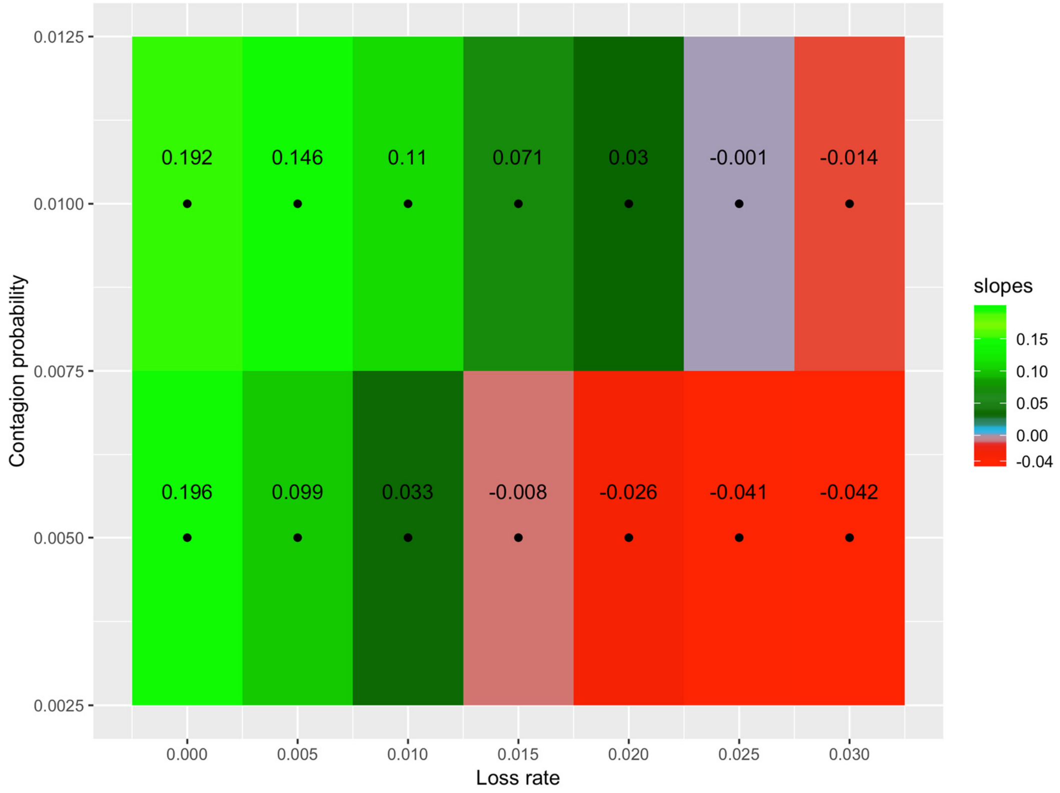

Figure S4.4. Effect of considering a ratio of 4 virulence genes for 1 resistance gene. Slope of the regression between the diversity of virulence genes and resistance genes according to the contagion probability (vertical axis) and the loss rate (horizontal axis). Green: positive slopes; and red: negative slopes.

6 – The correlation's sign is robust under changes in the gene elimination probability when people take antibiotics (considering equal probabilities to eliminate virulence and resistance genes)

Table S5.1. The impact of considering a probability of eliminating genes under antibiotic intake of 30% on the correlation between virulence and resistance genes.

Contagion probability (%)	Resistance genes loss rate (%)	Slope considering a probability of 30%	Slope considering a probability of 70%	P-value of T-test for differences of slopes	Change in the slope sign?
**0.5**	0	0.663	0.775	3.16×10^{-19}	No
	0.5	0.303	0.381	4.48×10^{-7}	No
	1	0.030	0.109	1.97×10^{-6}	No
	1.5	-0.138	-0.038	6.21×10^{-12}	No
	2	-0.245	-0.113	7.68×10^{-22}	No
	2.5	-0.288	-0.145	3.29×10^{-27}	No
	3	-0.314	-0.174	1.40×10^{-34}	No
**1**	0	0.712	0.742	2.66×10^{-3}	No
	0.5	0.540	0.586	6.46×10^{-3}	No
	1	0.354	0.431	3.44×10^{-5}	No
	1.5	0.250	0.253	8.89×10^{-1}	No
	2	0.011	0.106	1.60×10^{-5}	No
	2.5	-0.128	-0.002	2.86×10^{-11}	No
	3	-0.227	-0.069	2.20×10^{-17}	No

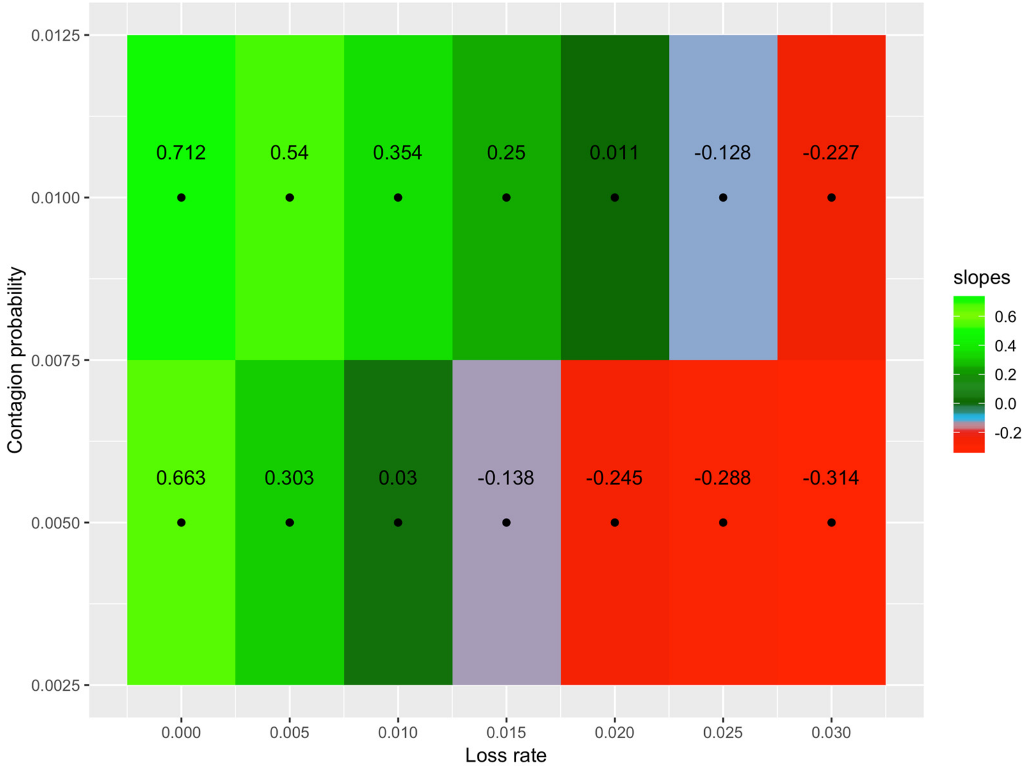

Figure S5.1. Effect of considering a probability of eliminating genes under antibiotic intake of 30%. Slope of the regression between the diversity of virulence genes and resistance genes according to the contagion probability (vertical axis) and the loss rate (horizontal axis). Green: positive slopes; and red: negative slopes.

Table S5.2. The impact of considering a probability of eliminating genes under antibiotic intake of 50% on the correlation between virulence and resistance genes.

Contagion probability (%)	Resistance genes loss rate (%)	Slope considering a probability of 50%	Slope considering a probability of 70%	P-value of T-test for differences of slopes	Change in the slope sign?
**0.5**	0	0.697	0.775	1.33×10^{-9}	No
	0.5	0.359	0.381	7.26×10^{-2}	No
	1	0.091	0.109	1.14×10^{-1}	No
	1.5	-0.072	-0.038	2.00×10^{-3}	No
	2	-0.144	-0.113	2.88×10^{-3}	No
	2.5	-0.189	-0.145	1.07×10^{-5}	No
	3	-0.195	-0.174	1.34×10^{-2}	No
**1**	0	0.723	0.742	4.60×10^{-2}	No
	0.5	0.583	0.586	7.43×10^{-1}	No
	1	0.379	0.431	1.97×10^{-4}	No
	1.5	0.269	0.253	3.09×10^{-1}	No
	2	0.094	0.106	4.16×10^{-1}	No
	2.5	-0.037	-0.002	9.49×10^{-3}	No
	3	-0.113	-0.069	1.32×10^{-4}	No

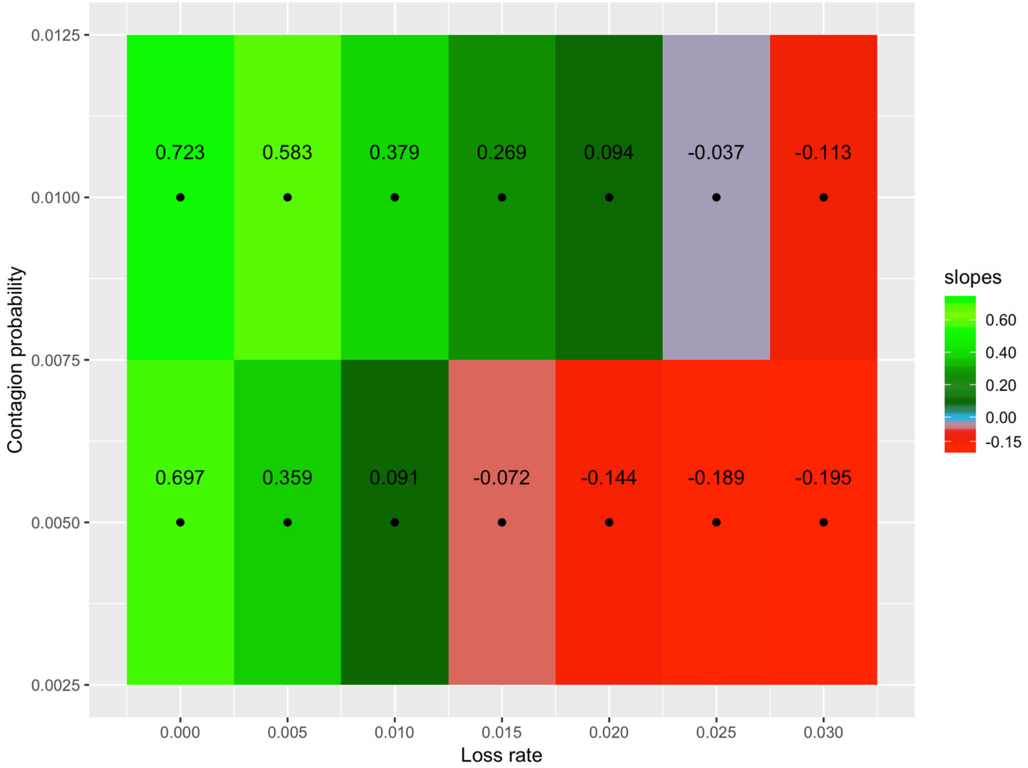

Figure S5.2. Effect of considering a probability of eliminating genes under antibiotic intake of 50%. Slope of the regression between the diversity of virulence genes and resistance genes according to the contagion probability (vertical axis) and the loss rate (horizontal axis). Green: positive slopes; and red: negative slopes.

7 – The correlation's sign is robust under changes in the gene elimination probability when people take antibiotics (considering different probabilities to eliminate virulence and resistance genes)

Table S6.1. The impact of considering a probability of eliminating virulence genes under antibiotic intake of 30% and a probability of eliminating resistance genes under antibiotic intake of 50% on the correlation between virulence and resistance genes.

Contagion probability (%)	Resistance genes loss rate (%)	Slope considering a probability of 30% for virulence genes and 50% for resistance genes	Slope considering a probability of 70%	P-value of T-test for differences of slopes	Change in the slope sign?
**0.5**	0	0.943	0.775	4.23×10^{-26}	No
	0.5	0.500	0.381	6.64×10^{-15}	No
	1	0.135	0.109	1.05×10^{-1}	No
	1.5	-0.100	-0.038	1.06×10^{-5}	No
	2	-0.210	-0.113	6.56×10^{-15}	No
	2.5	-0.244	-0.145	7.54×10^{-17}	No
	3	-0.272	-0.174	1.05×10^{-18}	No
**1**	0	1.110	0.742	8.33×10^{-137}	No
	0.5	0.852	0.586	4.45×10^{-56}	No
	1	0.645	0.431	3.50×10^{-24}	No
	1.5	0.355	0.253	2.67×10^{-6}	No
	2	0.155	0.106	1.85×10^{-2}	No
	2.5	-0.048	-0.002	9.15×10^{-3}	No
	3	-0.183	-0.069	6.14×10^{-11}	No

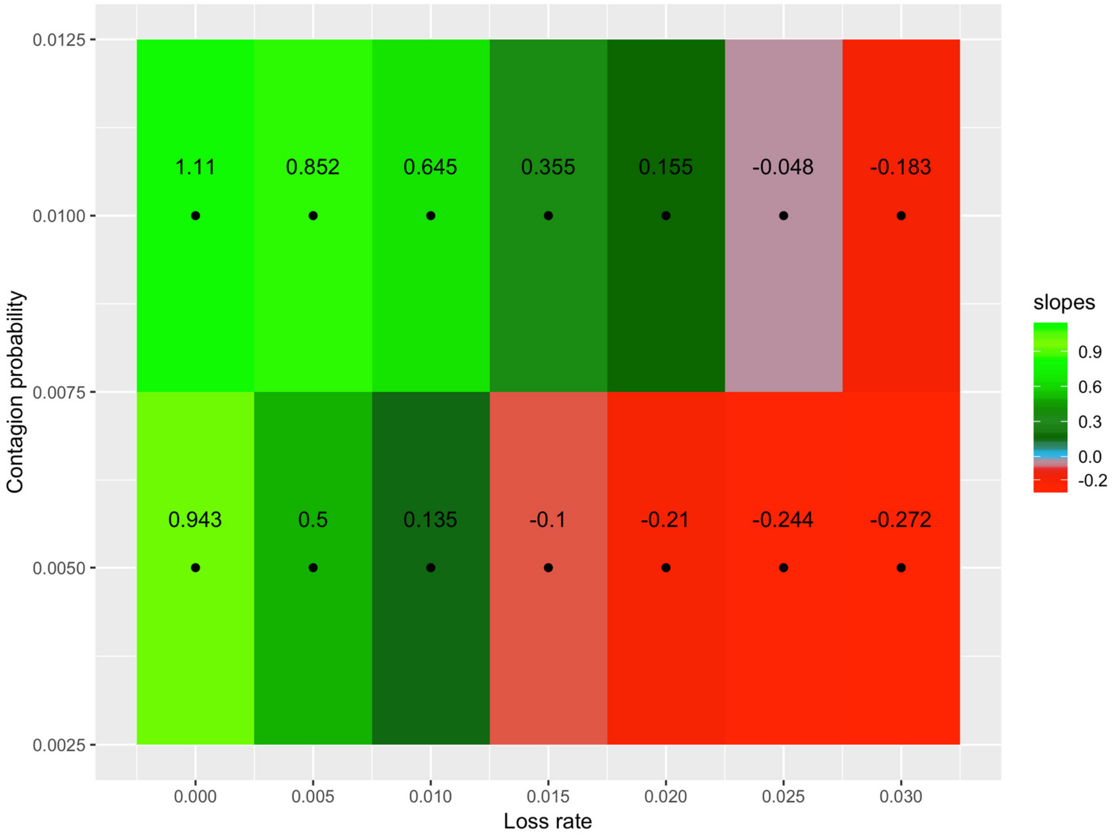

Figure S6.1. Effect of considering a probability of eliminating virulence genes under antibiotic intake of 30% and a probability of eliminating resistance genes under antibiotic intake of 50%. Slope of the regression between the diversity of virulence genes and resistance genes according to the contagion probability (vertical axis) and the loss rate (horizontal axis). Green: positive slopes; and red: negative slopes.

Table S6.2. The impact of considering a probability of eliminating virulence genes under antibiotic intake of 30% and a probability of eliminating resistance genes under antibiotic intake of 70% on the correlation between virulence and resistance genes.

Contagion probability (%)	Resistance genes loss rate (%)	Slope considering a probability of 30% for virulence genes and 70% for resistance genes	Slope considering a probability of 70%	P-value of T-test for differences of slopes	Change in the slope sign?
**0.5**	0	1.115	0.775	$2.00x10^{-65}$	No
	0.5	0.595	0.381	$4.88x10^{-39}$	No
	1	0.161	0.109	$2.19x10^{-4}$	No
	1.5	-0.058	-0.038	$1.12x10^{-1}$	No
	2	-0.167	-0.113	$3.09x10^{-6}$	No
	2.5	-0.217	-0.145	$3.12x10^{-11}$	No
	3	-0.234	-0.174	$6.32x10^{-9}$	No
**1**	0	1.466	0.742	$4.11x10^{-271}$	No
	0.5	1.078	0.586	$1.76x10^{-141}$	No
	1	0.838	0.431	$1.03x10^{-68}$	No
	1.5	0.419	0.253	$7.25x10^{-17}$	No
	2	0.252	0.106	4.1110^{-12}	No
	2.5	0.016	-0.002	$3.00x10^{-1}$	Yes
	3	-0.100	-0.069	$3.11x10^{-2}$	No

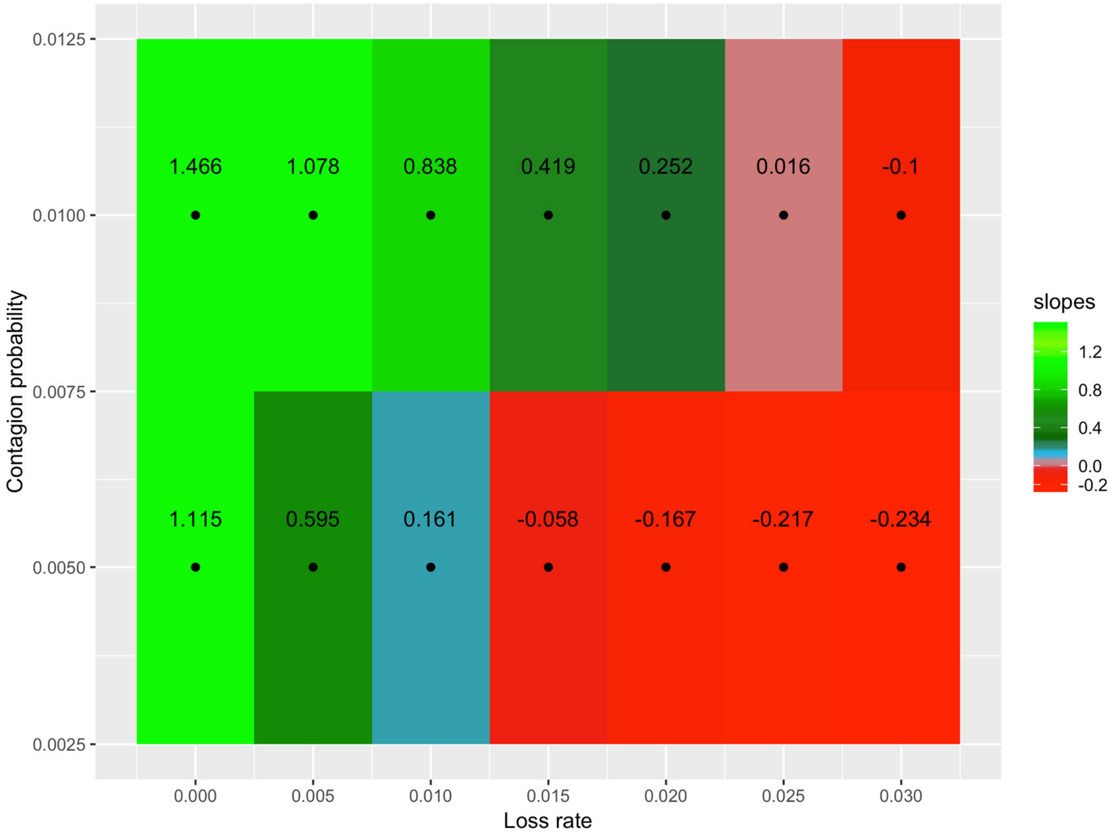

Figure S6.2. Effect of considering a probability of eliminating virulence genes under antibiotic intake of 30% and a probability of eliminating resistance genes under antibiotic intake of 70%. Slope of the regression between the diversity of virulence genes and resistance genes according to the contagion probability (vertical axis) and the loss rate (horizontal axis). Green: positive slopes; and red: negative slopes.

**Table S6.3.** The impact of considering a probability of eliminating virulence genes under antibiotic intake of 50% and a probability of eliminating resistance genes under antibiotic intake of 30% on the correlation between virulence and resistance genes.					
**Contagion probability (%)**	**Resistance genes loss rate (%)**	**Slope considering a probability of 50% for virulence genes and 30% for resistance genes**	**Slope considering a probability of 70%**	**P-value of T-test for differences of slopes**	**Change in the slope sign?**
**0.5**	0	0.467	0.775	6.04×10^{-138}	No
	0.5	0.262	0.381	5.73×10^{-22}	No
	1	0.037	0.109	4.59×10^{-8}	No
	1.5	-0.111	-0.038	2.98×10^{-10}	No
	2	-0.192	-0.113	1.73×10^{-13}	No
	2.5	-0.224	-0.145	6.39×10^{-16}	No
	3	-0.231	-0.174	1.07×10^{-9}	No
**1**	0	0.465	0.742	3.90×10^{-199}	No
	0.5	0.360	0.586	9.46×10^{-81}	No
	1	0.277	0.431	5.37×10^{-25}	No
	1.5	0.110	0.253	2.06×10^{-20}	No
	2	0.024	0.106	1.51×10^{-7}	No
	2.5	-0.088	-0.002	2.98×10^{-9}	No
	3	-0.137	-0.069	2.60×10^{-8}	No

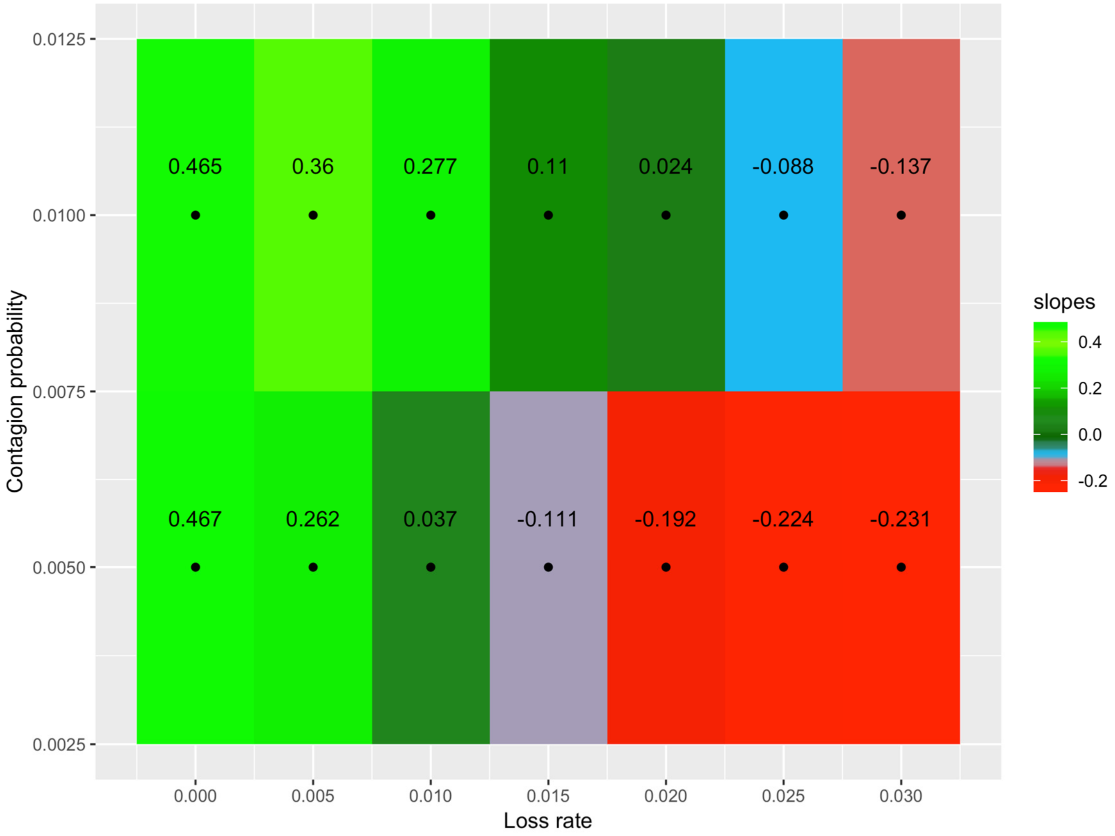

Figure S6.3. Effect of considering a probability of eliminating virulence genes under antibiotic intake of 50% and a probability of eliminating resistance genes under antibiotic intake of 30%. Slope of the regression between the diversity of virulence genes and resistance genes according to the contagion probability (vertical axis) and the loss rate (horizontal axis). Green: positive slopes; and red: negative slopes.

Table S6.4. The impact of considering a probability of eliminating virulence genes under antibiotic intake of 50% and a probability of eliminating resistance genes under antibiotic intake of 70% on the correlation between virulence and resistance genes.

**Contagion probability (%)**	**Resistance genes loss rate (%)**	**Slope considering a probability of 50% for virulence genes and 70% for resistance genes**	**Slope considering a probability of 70%**	**P-value of T-test for differences of slopes**	**Change in the slope sign?**
**0.5**	0	0.836	0.775	$8.71x10^{-6}$	No
	0.5	0.437	0.381	$4.09x10^{-6}$	No
	1	0.120	0.109	$3.44x10^{-1}$	No
	1.5	-0.041	-0.038	$7.61x10^{-1}$	No
	2	-0.121	-0.113	$3.90x10^{-1}$	No
	2.5	-0.173	-0.145	$1.27x10^{-3}$	No
	3	-0.174	-0.174	$9.56x10^{-1}$	No
**1**	0	0.945	0.742	$1.63x10^{-79}$	No
	0.5	0.739	0.586	$2.41x10^{-35}$	No
	1	0.521	0.431	$1.87x10^{-10}$	No
	1.5	0.346	0.253	$3.00x10^{-9}$	No
	2	0.151	0.106	$1.42x10^{-3}$	No
	2.5	0.016	-0.002	$1.50x10^{-1}$	Yes
	3	-0.068	-0.069	$9.16x10^{-1}$	No

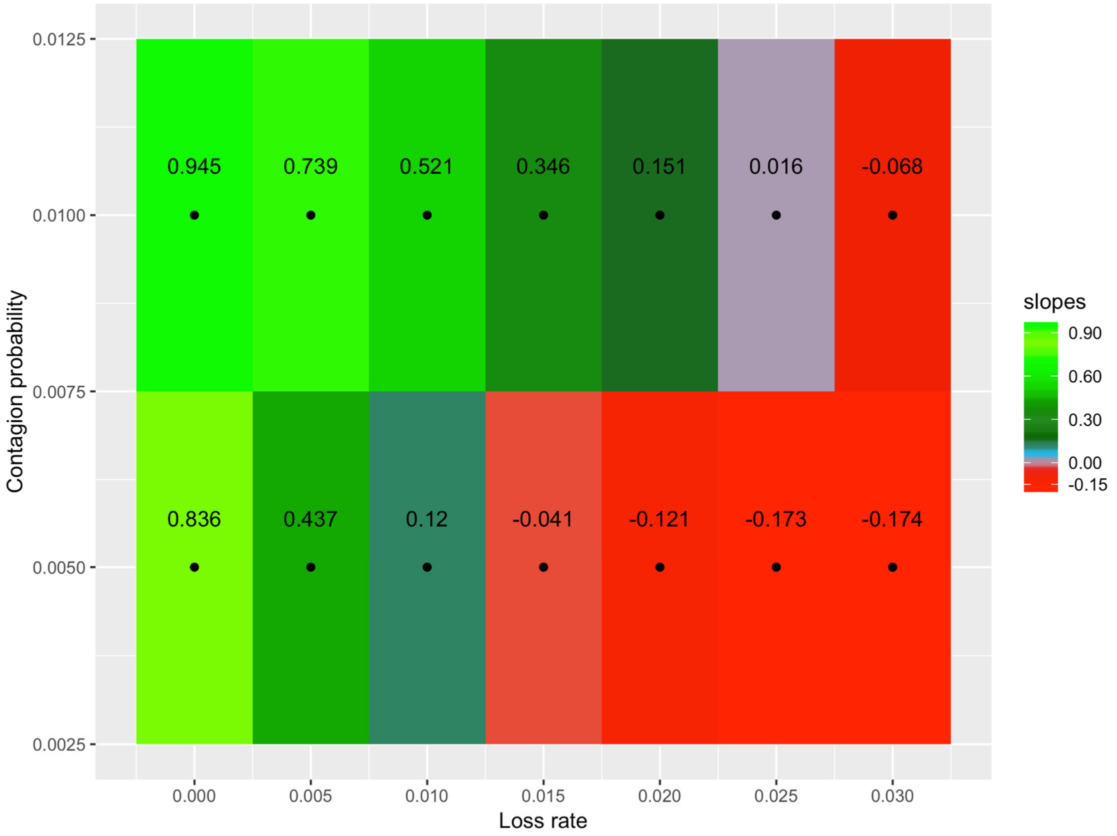

Figure S6.4. Effect of considering a probability of eliminating virulence genes under antibiotic intake of 50% and a probability of eliminating resistance genes under antibiotic intake of 70%. Slope of the regression between the diversity of virulence genes and resistance genes according to the contagion probability (vertical axis) and the loss rate (horizontal axis). Green: positive slopes; and red: negative slopes.

Table S6.5. The impact of considering a probability of eliminating virulence genes under antibiotic intake of 70% and a probability of eliminating resistance genes under antibiotic intake of 30% on the correlation between virulence and resistance genes.

Contagion probability (%)	Resistance genes loss rate (%)	Slope considering a probability of 70% for virulence genes and 30% for resistance genes	Slope considering a probability of 70%	P-value of T-test for differences of slopes	Change in the slope sign?
**0.5**	0	0.418	0.775	2.96×10^{-153}	No
	0.5	0.217	0.381	3.75×10^{-40}	No
	1	0.016	0.109	1.95×10^{-13}	No
	1.5	-0.101	-0.038	1.81×10^{-7}	No
	2	-0.189	-0.113	1.38×10^{-11}	No
	2.5	-0.182	-0.145	7.19×10^{-5}	No
	3	-0.195	-0.174	1.97×10^{-2}	No
**1**	0	0.364	0.742	0	No
	0.5	0.284	0.586	7.17×10^{-179}	No
	1	0.181	0.431	2.58×10^{-82}	No
	1.5	0.099	0.253	1.04×10^{-29}	No
	2	0.010	0.106	1.55×10^{-13}	No
	2.5	-0.060	-0.002	1.36×10^{-16}	No
	3	-0.135	-0.069	8.50×10^{-11}	No

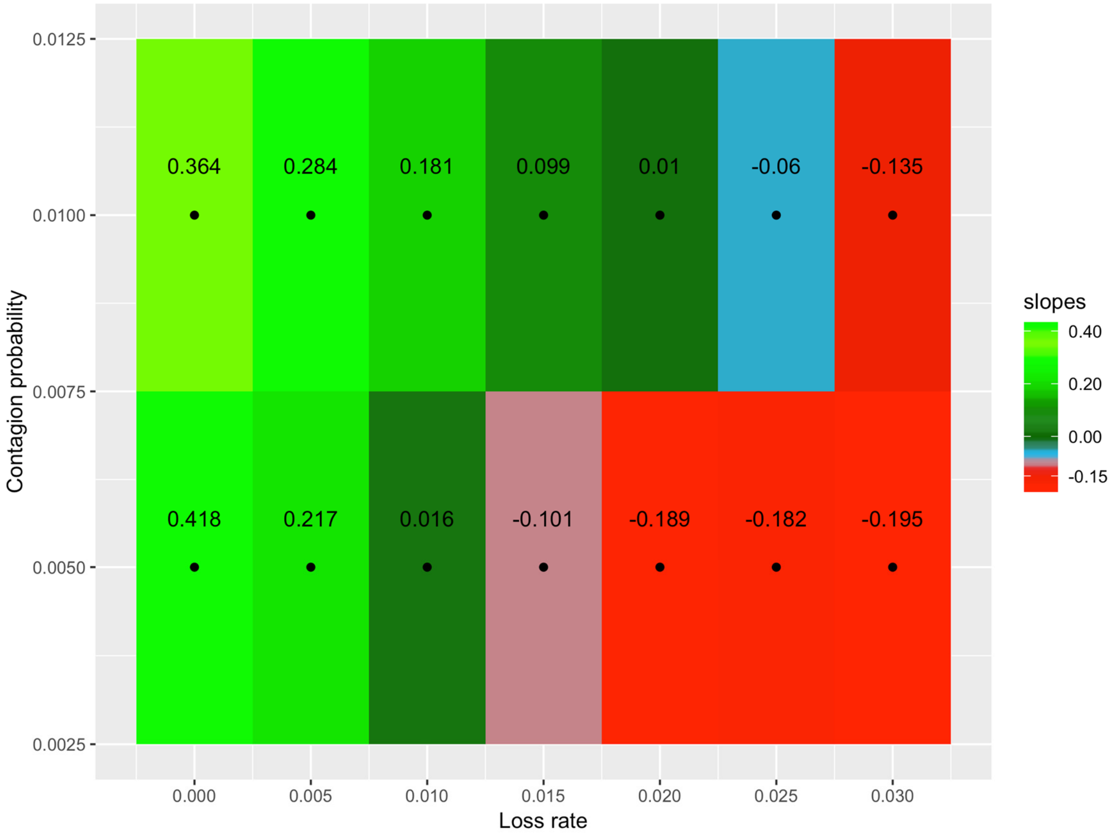

Figure S6.5. Effect of considering a probability of eliminating virulence genes under antibiotic intake of 70% and a probability of eliminating resistance genes under antibiotic intake of 30%. Slope of the regression between the diversity of virulence genes and resistance genes according to the contagion probability (vertical axis) and the loss rate (horizontal axis). Green: positive slopes; and red: negative slopes.

**Table S6.6.** The impact of considering a probability of eliminating virulence genes under antibiotic intake of 70% and a probability of eliminating resistance genes under antibiotic intake of 50% on the correlation between virulence and resistance genes.					
**Contagion probability (%)**	**Resistance genes loss rate (%)**	**Slope considering a probability of 70% for virulence genes and 50% for resistance genes**	**Slope considering a probability of 70%**	**P-value of T-test for differences of slopes**	**Change in the slope sign?**
**0.5**	0	0.627	0.775	5.91×10^{-29}	No
	0.5	0.351	0.381	1.44×10^{-2}	No
	1	0.087	0.109	5.40×10^{-2}	No
	1.5	-0.064	-0.038	1.61×10^{-2}	No
	2	-0.144	-0.113	1.43×10^{-3}	No
	2.5	-0.165	-0.145	3.39×10^{-2}	No
	3	-0.178	-0.174	6.10×10^{-1}	No
**1**	0	0.563	0.742	1.73×10^{-99}	No
	0.5	0.439	0.586	6.75×10^{-48}	No
	1	0.314	0.431	6.64×10^{-21}	No
	1.5	0.196	0.253	5.59×10^{-6}	No
	2	0.073	0.106	1.30×10^{-2}	No
	2.5	-0.033	-0.002	8.33×10^{-3}	No
	3	-0.094	-0.069	1.08×10^{-2}	No

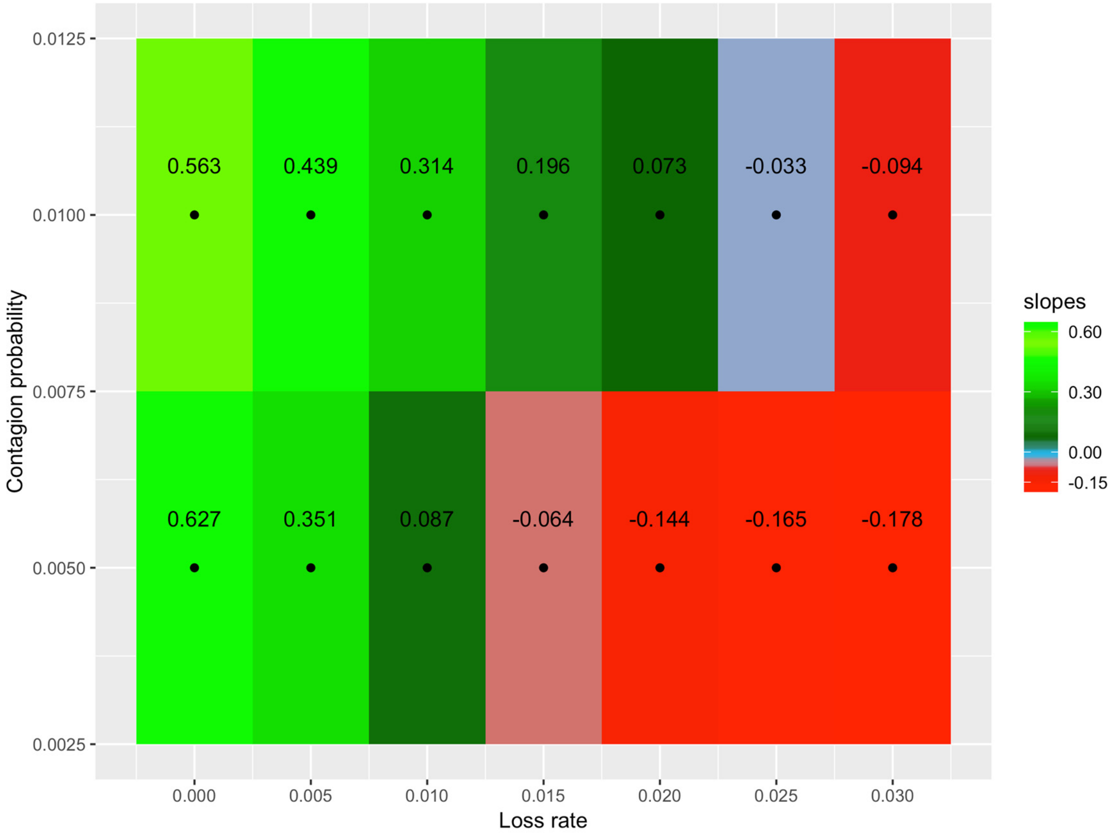

Figure S6.6. Effect of considering a probability of eliminating virulence genes under antibiotic intake of 70% and a probability of eliminating resistance genes under antibiotic intake of 50%. Slope of the regression between the diversity of virulence

genes and resistance genes according to the contagion probability (vertical axis) and the loss rate (horizontal axis). Green: positive slopes; and red: negative slopes.

8 – The initial proportion of metagenomes containing antibiotic resistance genes has no impact on correlations' sign

Table S7.1. The impact of considering that, initially, only 10% of metagenomes contain antibiotic resistance genes on the correlation between virulence and resistance genes.

Contagion probability (%)	Resistance genes loss rate (%)	Slope considering 10% metagenomes containing resistance genes initially	Slope considering all metagenomes containing resistance genes initially	P-value of T-test for differences of slopes	Change in the slope sign?
**0.5**	0	0.774	0.775	9.40×10^{-1}	No
	0.5	0.394	0.381	2.68×10^{-1}	No
	1	0.129	0.109	6.77×10^{-2}	No
	1.5	-0.038	-0.038	9.79×10^{-1}	No
	2	-0.113	-0.113	9.88×10^{-1}	No
	2.5	-0.146	-0.145	9.08×10^{-1}	No
	3	-0.157	-0.174	4.44×10^{-2}	No
**1**	0	0.748	0.742	4.66×10^{-1}	No
	0.5	0.586	0.586	9.34×10^{-1}	No
	1	0.423	0.431	4.84×10^{-1}	No
	1.5	0.270	0.253	1.79×10^{-1}	No
	2	0.119	0.106	2.86×10^{-1}	No
	2.5	0.006	-0.002	4.40×10^{-1}	Yes
	3	-0.058	-0.069	2.55×10^{-1}	No

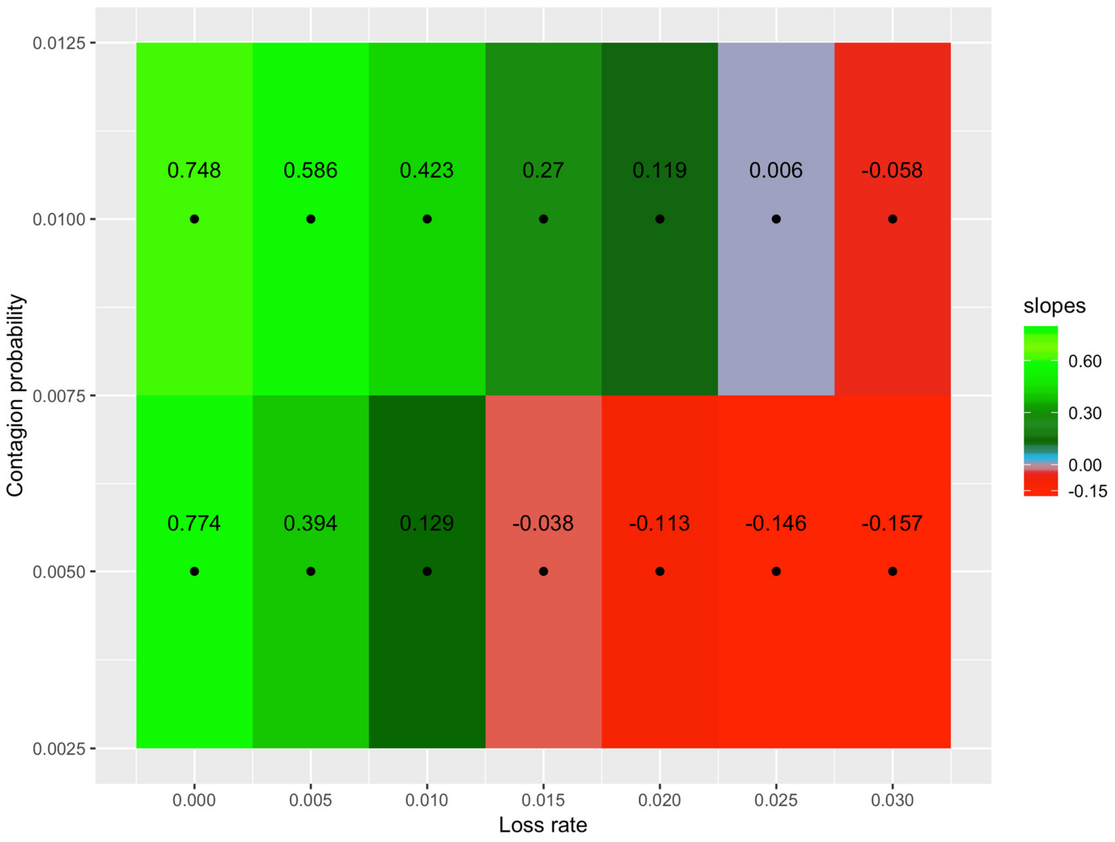

Figure S7.1. Effect of considering that, initially, only 10% of metagenomes contain antibiotic resistance genes. Slope of the regression between the diversity of virulence genes and resistance genes according to the contagion probability (vertical axis) and the loss rate (horizontal axis). Green: positive slopes; and red: negative slopes.

9 – The network type has no impact on correlation's sign

**Table S8.1.** The impact of considering a random network (p=1) on the correlation between virulence and resistance genes.					
**Contagion probability (%)**	**Resistance genes loss rate (%)**	**Slope considering a random network (p=1)**	**Slope considering a small-world network (p=0.5)**	**P-value of T-test for differences of slopes**	**Change in the slope sign?**
**0.5**	0	0.789	0.775	3.10×10^{-1}	No
	0.5	0.425	0.381	1.57×10^{-4}	No
	1	0.105	0.109	6.59×10^{-1}	No
	1.5	-0.014	-0.038	2.77×10^{-2}	No
	2	-0.113	-0.113	9.78×10^{-1}	No
	2.5	-0.150	-0.145	6.17×10^{-1}	No
	3	-0.149	-0.174	5.59×10^{-3}	No
**1**	0	0.745	0.742	7.47×10^{-1}	No
	0.5	0.575	0.586	2.40×10^{-1}	No
	1	0.412	0.431	1.24×10^{-1}	No
	1.5	0.272	0.253	1.31×10^{-1}	No
	2	0.133	0.106	3.21×10^{-2}	No
	2.5	0.023	-0.002	2.06×10^{-2}	Yes
	3	-0.059	-0.069	3.13×10^{-1}	No

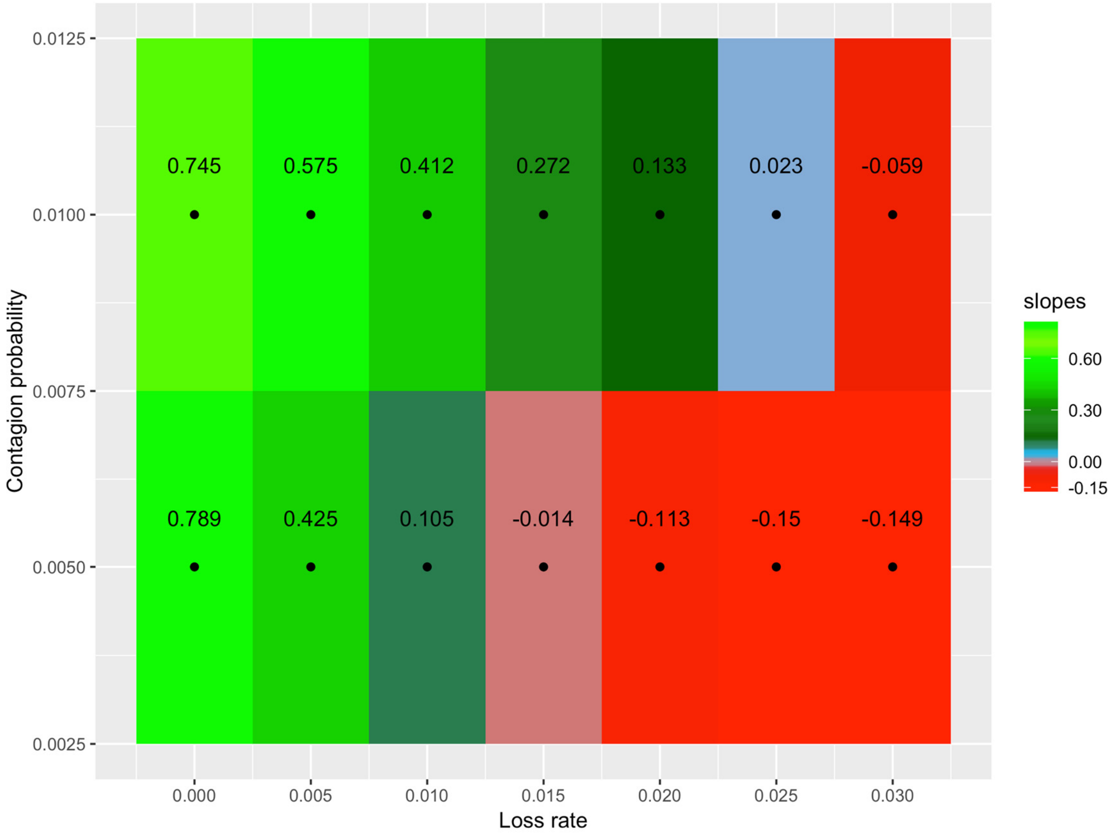

Figure S8.1. Effect of considering a random network (p=1). Slope of the regression between the diversity of virulence genes and resistance genes according to the contagion probability (vertical axis) and the loss rate (horizontal axis). Green: positive slopes; and red: negative slopes.

Table S8.2. The impact of considering a regular network (p=0) on the correlation between virulence and resistance genes.

Contagion probability (%)	Resistance genes loss rate (%)	Slope considering a regular network (p=0)	Slope considering a small-world network (p=0.5)	P-value of T-test for differences of slopes	Change in the slope sign?
	0	7.062	0.775	7.48x10^{-38}	No
	0.005	2.589	0.381	3.31x10^{-21}	No
	0.01	0.569	0.109	1.23x10^{-4}	No
**0.5**	0.015	-0.93	-0.038	1.46x10^{-11}	No
	0.02	-1.135	-0.113	1.23x10^{-16}	No
	0.025	-1.304	-0.145	4.17x10^{-25}	No
	0.03	-1.076	-0.174	1.81x10^{-16}	No
	0	0.729	0.742	1.39x10^{-1}	No
	0.005	0.602	0.586	8.97x10^{-2}	No
	0.01	0.394	0.431	6.77x10^{-4}	No
**1**	0.015	0.193	0.253	3.66x10^{-8}	No
	0.02	0.02	0.106	5.90x10^{-17}	No
	0.025	-0.08	-0.002	8.76x10^{-17}	No
	0.03	-0.12	-0.069	1.86x10^{-10}	No

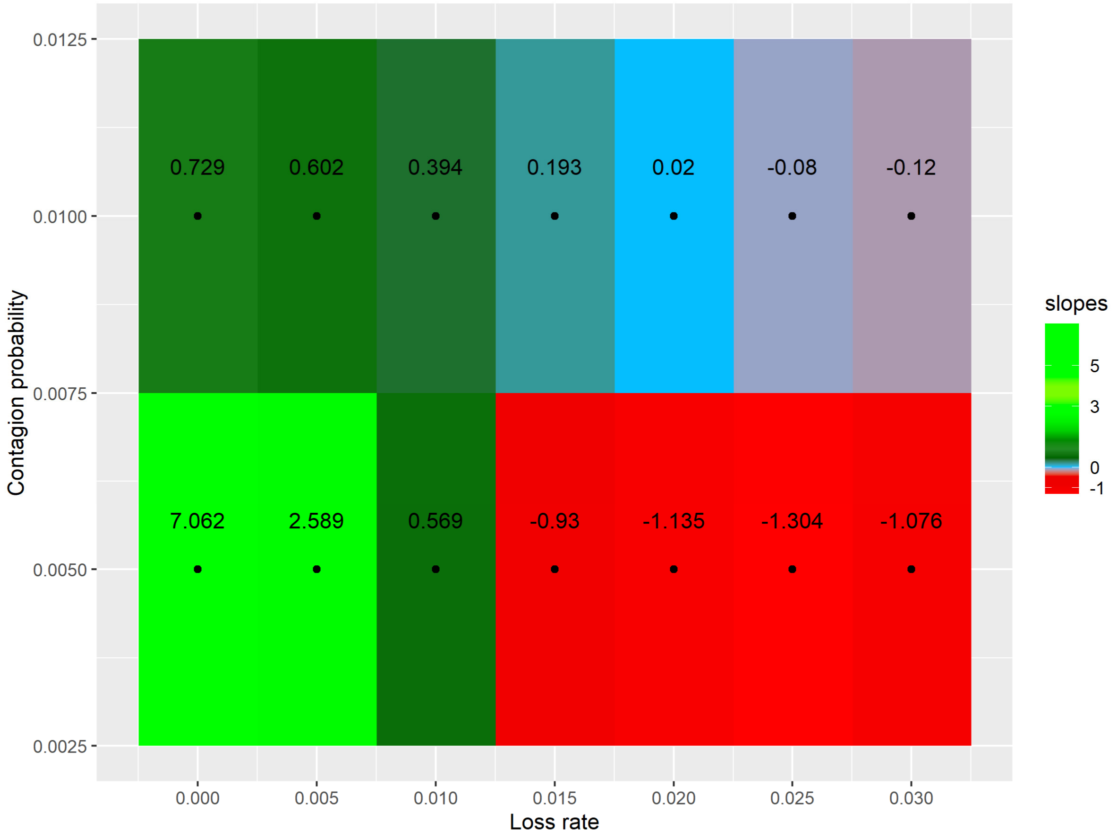

Figure S8.2. Effect of considering a regular network (p=0). Slope of the regression between the diversity of virulence genes and resistance genes according to the contagion probability (vertical axis) and the loss rate (horizontal axis). Green: positive slopes; and red: negative slopes.

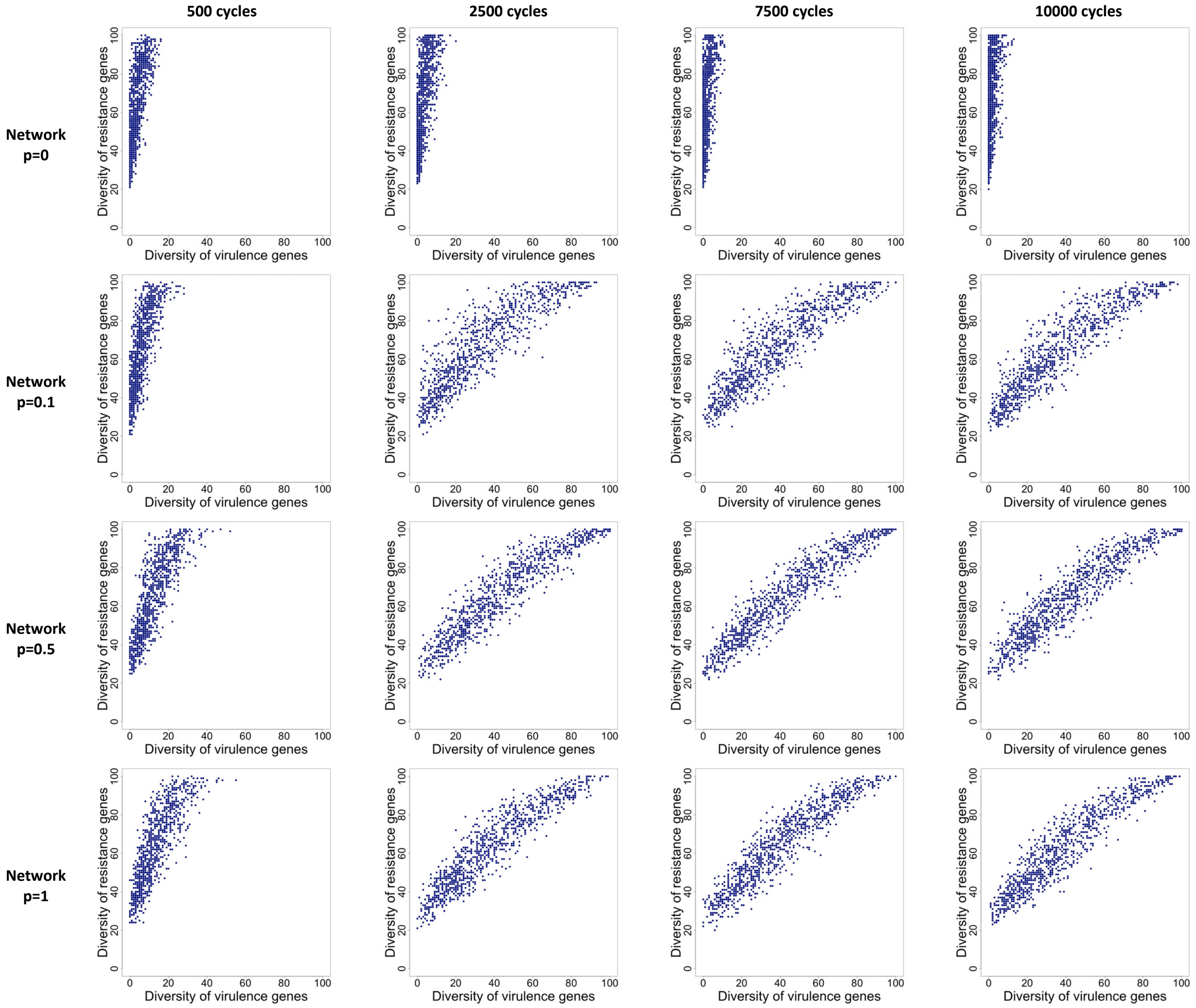

Figure S8.3. Effect of the networks. The correlations obtained using a network with p = 0.5 or 1 are stable at cycle 2500, while the correlation obtained with network p = 0.1 is only stable at cycle 7500. Using a network with p = 0, there is no correlation because there is loss of diversity of virulence genes. Correlations obtained for contagion probability of 0.5% and loss rate of 0%. Columns: (1) Cycle 500; (2) Cycle 2500; (3) Cycle 7500; and (4) Cycle 10000. Rows: (1) p=0; (2) p=0.1; (3) p=0.5; and (4) p=1. The vertical axes represent the diversity of resistance genes. The horizontal axes represent the diversity of virulence genes. Each dot represents the diversity of virulence genes present in an individual metagenome. The results were obtained using the parameters described in Methods (Table 2).
